# Crystallographic cyanide-probing for cytochrome *c* oxidase reveals structural bases suggesting that a putative proton transfer H-pathway pumps protons

**DOI:** 10.1016/j.jbc.2023.105277

**Published:** 2023-09-22

**Authors:** Atsuhiro Shimada, Jumpei Baba, Shuhei Nagao, Kyoko Shinzawa-Itoh, Eiki Yamashita, Kazumasa Muramoto, Tomitake Tsukihara, Shinya Yoshikawa

**Affiliations:** 1 Picobiology Institute, Graduate School of Life Science, University of Hyogo, Hyogo, Japan; 2 Department of Life Science, Graduate School of Life Science, University of Hyogo, Kamigori, Akoh, Hyogo, Japan; 3 Institute for Protein Research, Osaka University, Suita, Osaka, Japan

**Keywords:** cytochrome *c* oxidase, proton pump, O_2_-reduction, hemoprotein, cyanide inhibition, X-ray crystallographic analysis

## Abstract

Cytochrome *c* oxidase (CcO) reduces O_2_ in the O_2_-reduction site by sequential four-electron donations through the low-potential metal sites (Cu_A_ and Fe_*a*_). Redox-coupled X-ray crystal structural changes have been identified at five distinct sites including Asp^51^, Arg^438^, Glu^198^, the hydroxyfarnesyl ethyl group of heme *a*, and Ser^382^, respectively. These sites interact with the putative proton-pumping H-pathway. However, the metal sites responsible for each structural change have not been identified, since these changes were detected as structural differences between the fully reduced and fully oxidized CcOs. Thus, the roles of these structural changes in the CcO function are yet to be revealed. X-ray crystal structures of cyanide-bound CcOs under various oxidation states showed that the O_2_-reduction site controlled only the Ser^382^-including site, while the low-potential metal sites induced the other changes. This finding indicates that these low-potential site-inducible structural changes are triggered by sequential electron-extraction from the low-potential sites by the O_2_-reduction site and that each structural change is insensitive to the oxidation and ligand-binding states of the O_2_-reduction site. Because the proton/electron coupling efficiency is constant (1:1), regardless of the reaction progress in the O_2_-reduction site, the structural changes induced by the low-potential sites are assignable to those critically involved in the proton pumping, suggesting that the H-pathway, facilitating these low-potential site-inducible structural changes, pumps protons. Furthermore, a cyanide-bound CcO structure suggests that a hypoxia-inducible activator, Higd1a, activates the O_2_-reduction site without influencing the electron transfer mechanism through the low-potential sites, kinetically confirming that the low-potential sites facilitate proton pump.

Mammalian cytochrome *c* oxidase (CcO), as the terminal oxidase of cell respiration, reduces molecular oxygen (O_2_) to water, coupled with a proton pump across the enzyme molecule from the matrix side (N-side) to the intermembrane side (P-side) to create a proton gradient across the mitochondrial inner membrane (1, 2, 3). Electrons for reducing O_2_ are transferred from cytochrome *c* in the P-side to the O_2_-reduction site, including heme *a*_3_ (Fe_*a*3_) and Cu_B_ *via* the two low-potential sites, Cu_A_ and heme *a* (Fe_*a*_), while protons for making water molecules from the fully reduced O_2_ (*i.e.*, 2O^2-^) to 2H_2_O are transferred to the O_2_-reduction site from the N-side *via* two proton-conducting pathways, D and K (1, 2, 3) as described in Figure 1. The electron and proton transfers create membrane potentials on the mitochondrial inner membrane. The resultant proton motive force (pmf), composed of the proton gradient and membrane potential, drives ATP formation by F-ATP synthase (1, 2, 3). The location of Tyr^244^ near the O_2_-reduction site, as revealed by X-ray structural analysis, strongly suggests that the OH group of Tyr^244^ donates a hydrogen atom to the O_2_ bound at Fe_*a*3_, forming a neutral oxygen radical (Tyr^244^O•) (1, 2, 4). The catalytic cycle of CcO includes six intermediate forms as follows, R (Fe_*a*3_^2+^, Cu_B_^1+^, Tyr^244^OH), A (Fe_*a*3_^2+^-O_2_, Cu_B_^1+^, Tyr^244^OH), P_m_ (Fe_*a*3_^4+^=O^2-^, Cu_B_^2+^-OH^-^, Tyr^244^O•), F (Fe_*a*3_^4+^=O^2-^, Cu_B_^2+^-OH^-^, Tyr^244^OH), O (Fe_*a*3_^3+^-OH^-^, Cu_B_^2+^-OH^-^, Tyr^244^OH), and E (Fe_*a*3_^3+^-OH^-^, Cu_B_^1+^-H_2_O, Tyr^244^OH). In each of the four transitions, P_m_→F, F→O, O→E, and E→R, one electron and one proton for making water are transferred from the P-side and the N-side, respectively, coupled with pumping one proton (1, 2, 4).

**Figure 1:**
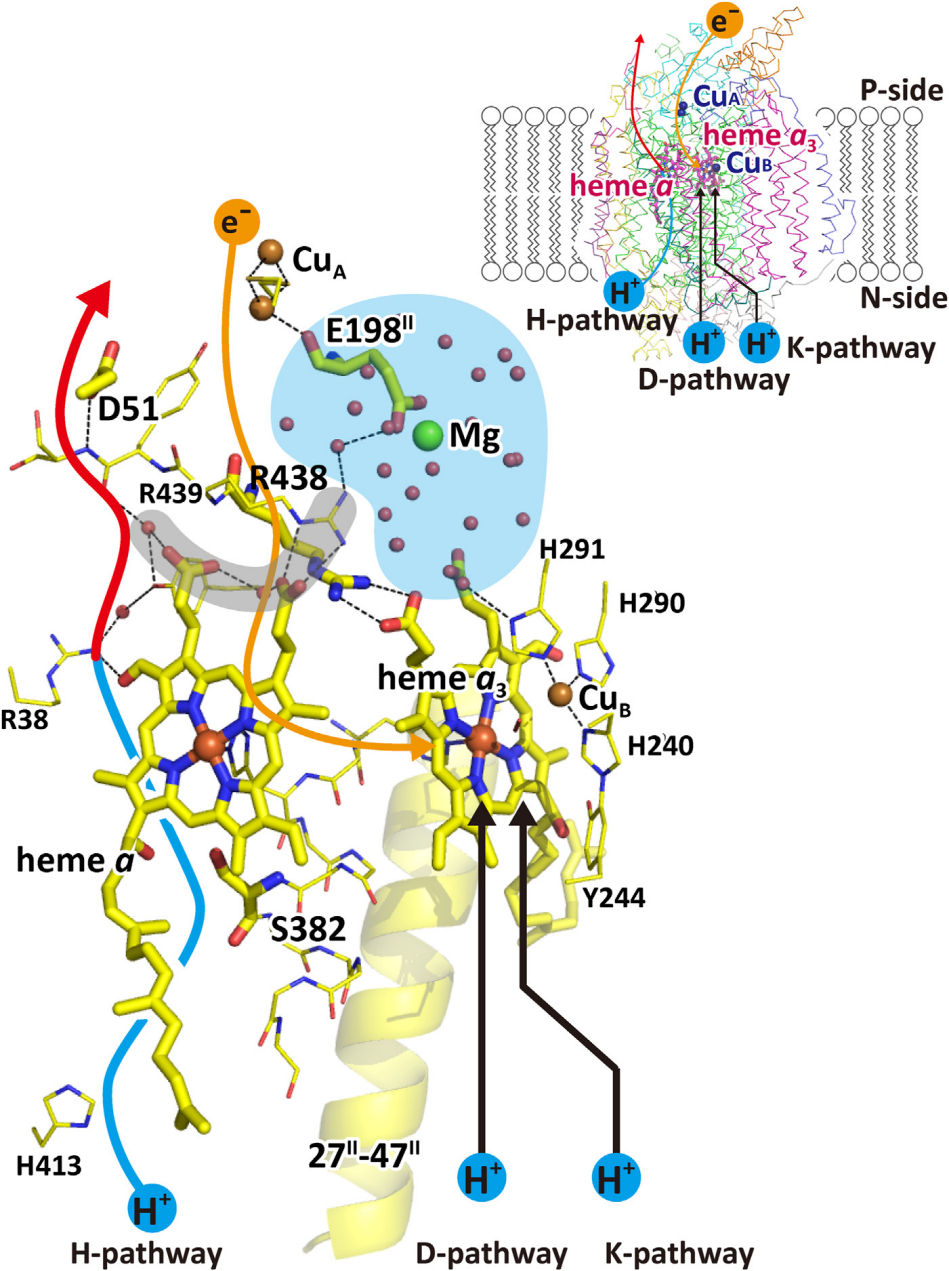
**X-ray structure of the active sites of bovine heart cytochrome *c* oxidase.** Metal sites are indicated by *brown*, *beige*, and *green* spheres for iron, copper, and magnesium ions, respectively. Porphyrins of heme *a* and heme *a*_3_ and amino acid residues showing redox-coupled conformational changes are represented by the *thick stick* models as labeled. Within the stick models, *dark blue*, *red*, and *yellow* portions are nitrogen, oxygen, and carbon, respectively. A *beige* arrow indicates the location of the electron transfer passage, while two *black* arrows indicate those for protons for producing water molecules. The hydrogen-bond network and the water channel of the H-pathway are indicated by the *red* and *blue* portions of the leftmost curved arrow, respectively. The Mg/H_2_O cluster (the *blue* area) is attached to the hydrogen-bond network of the H-pathway *via* a short hydrogen-bond network (the *gray* area). Small *beige* spheres mark the water molecule positions. The formyl group and one of the propionate groups of heme *a* are hydrogen-bonded with Arg^38^ and a fixed water molecule in the hydrogen-bond network of the H-pathway, respectively. The location of the residues 27-47 of one of the transmembrane helices of subunit II is shown by a *yellow* ribbon model as labeled. The inset shows the overall locations of the redox-active metal sites and pathways for transportation of electrons and protons within the CcO structure, indicated by Cα-backbone traces. This figure was prepared from the X-ray diffraction data of PDBID: 5B1A. CcO, cytochrome c oxidase.

Wikström’s group has determined that in each of the above four transitions driven by the proton-coupled electron transfer, one proton is pumped across the CcO molecule (5). This constant energy coupling seems quite unreasonable, since the chemical processes, each driving one of the four transitions, are clearly different from each other. For example, reduction of the Tyr^244^O radical occurs only in the P_m_→F transition. The constant (1:1) pumped proton to electron ratio should be attained by long and extensive trials and errors during biological evolution for improving the efficiency of the overall energy transduction between O_2_ reduction and pmf formation. Thus, identification of structural bases for constant energy coupling in the above four transitions would provide various critical insights for understanding the proton-pump mechanism of CcO.

The proton pump is driven by electrostatic interactions between the protons to be pumped and the net positive charges created during the O_2_ reduction. Two different proton pump mechanisms, each including either the D-pathway or H-pathway as the proton pumping site, were proposed about 30 years ago and are still under serious debate (1, 2, 3). The H-pathway is the third proton conducting pathway spanning across the CcO molecule from the N-side to the P-side composed of a water channel and a hydrogen bond network in tandem as marked by a blue and red arrow in Figure 1. A summary on the debate for the two proton-pumping proposals is given in Supporting information (Supporting Text 1 and Fig. S1).

The bovine heart CcO, as isolated from bovine heart muscle aerobically, is called the resting-oxidized form since the isolated CcO, in which all the metal sites are in the oxidized state, does not have a proton pumping function, in contrast to the O-form as described above (1, 2, 6). This form has a peroxide as the bridging ligand between Fe_*a*3_ and Cu_B_ instead of two OH^-^ groups in the O-form (6, 7). For the sake of simplicity, the resting-oxidized form is designated as the fully-oxidized form (hyphened between “fully” and “oxidized”) in this paper. Comparison of the structure of the fully reduced form with that of the fully-oxidized form showed five distinct redox-coupled structural changes, which interact with the H-pathway as described below (4, 8, 9, 10).

(1) The residues 48–55, including Asp^51^, located near the P-side end of the H-pathway. The redox-coupled structural changes schematically illustrated in Figure 2*A* suggest that Asp^51^ receives proton from the H-pathway in the oxidized state and releases it to the P-side in the reduced state, giving the active proton transport to the P-side.

**Figure 2:**
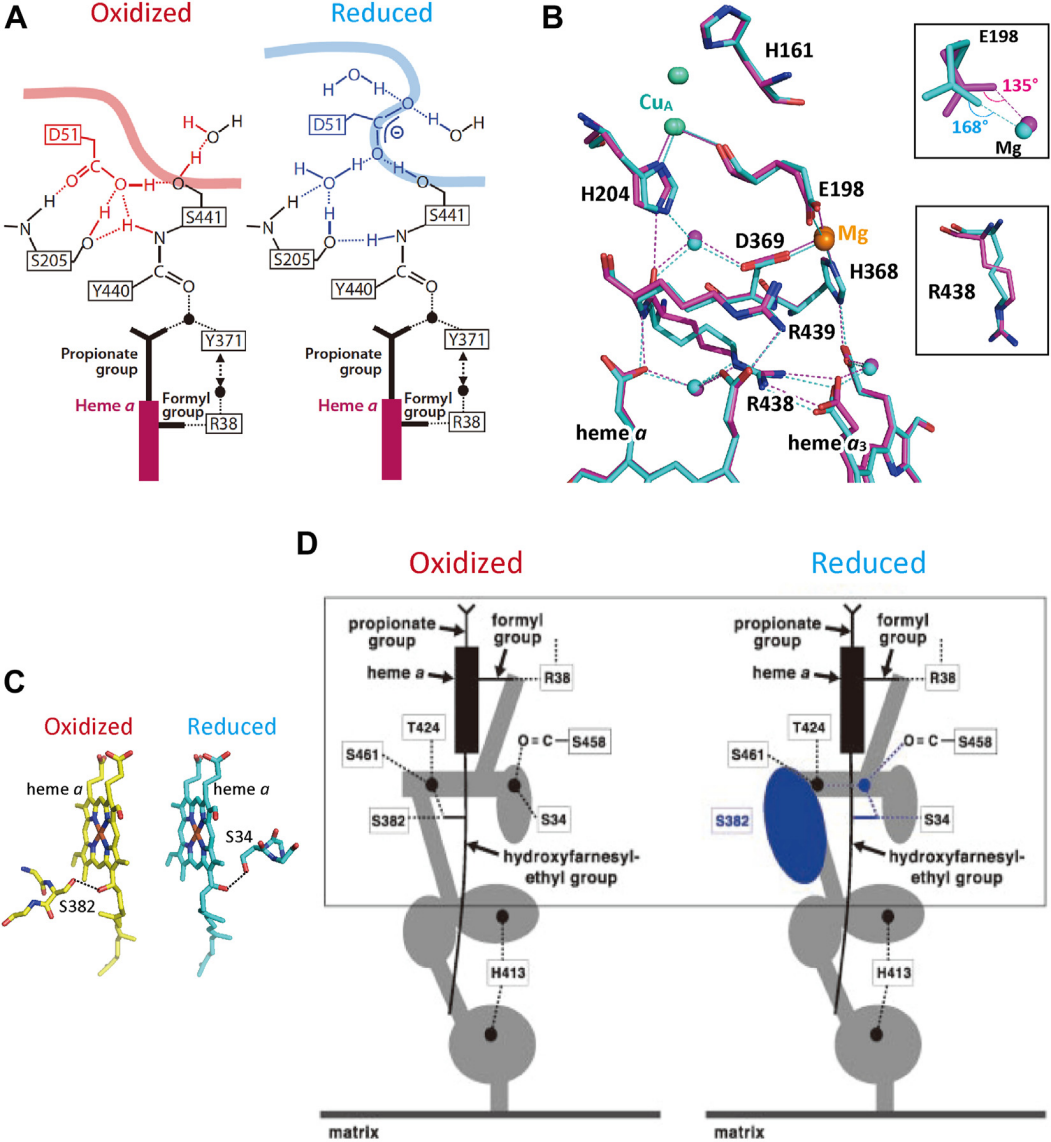
**Previously reported redox-coupled X-ray structural changes**. *A*, schematic representation of Asp^51^ in the fully-oxidized form structure (*left*) and the fully reduced form structure (*right*). The smooth thick curves denote the molecular surface accessible for the water molecules in the P-side phase. Reprinted with permission from Ref. (1). *B*, the redox-coupled structural changes in the region including Cu_A_ and Mg^2+^ site and four heme propionates. The *purple* and *blue* structures indicate those in the fully-oxidized and fully reduced form structures. Insets show magnified views of the redox-coupled structural changes at Glu^198^ and at Arg^438^. Reprinted with permission from Ref. (10). *C*, the structure of the hydroxyfarnesyl ethyl group in the fully-oxidized form and fully reduced form structures (marked in *yellow* and *blue*, respectively). Ser^382^ and Ser^34^ are hydrogen-bonded to the OH group of the hydroxyfarnesyl ethyl group in the fully-oxidized and fully reduced form structures, respectively. *D*, a schematic representation of the redox-coupled structural changes in the water channel detectable in the residues 380-385 region including Ser^382^. The water cavity colored in *blue* in the fully reduced form (*right*) is eliminated upon complete oxidation to the fully-oxidized form (*left*). Reprinted with permission from Ref. (1).
(2) Arg^438^. For effective proton pumping, appropriate control of duration of the reduced state of heme *a* during the catalytic cycle is required (11). It has been shown that heme *a* reduction significantly increases the redox potential of heme *a*_3_ to allow electron transfer from heme *a* to heme *a*_3_ (1, 2, 12). The guanidino group of Arg^438^ is salt bridged to one of the propionate groups of heme *a*_3_, while the main-chain portion of Arg^438^ is included in the electron transfer pathway from Cu_A_ to heme *a* as described in Figure 2*B* (10). Thus, the redox-coupled conformational changes in Arg^438^ (Fig. 2*B* lower inset) are likely to mediate the interaction between the two hemes for control of the oxidation state of the heme *a* for effective proton pumping.
(3) Glu^198^ of subunit II. Glu^198^ bridges between a binuclear copper site, Cu_A_, and a Mg^2+^ site in a large water cluster connected to the H-pathway as illustrated in Figures 1 and 2*B* (10). The main chain C=O of Glu^198^ is coordinated to the copper ions while the terminal carboxyl group is coordinated to the Mg^2+^ ion. The redox-coupled structural change in the residue, shown in Figure 2*B* upper inset, suggests a critical role of the water cluster for the storage and the timely release of the pumping proton to the H-pathway (10).
(4) The hydroxyfarnesyl ethyl group of heme *a.* It has been suggested that the proton pump of CcO is triggered by an electrostatic repulsion between protons on the hydrogen-bond network of the H-pathway and the net positive charges created upon oxidation of heme *a* (1, 3). In fact, the hydrogen-bond network of the H-pathway is attached to heme *a* by forming two hydrogen bonds with the formyl group and one of the propionate groups of heme *a*, as illustrated in Figure 1. Thus, the location of heme *a* relative to the hydrogen-bond network is critical for maximizing the proton pump efficiency. It has been shown that the OH group of hydroxylfarnesyl ethyl group of heme *a* forms a hydrogen bond with Ser^382^ and Ser^34^ in the fully oxidized and fully reduced states, respectively (Fig. 2*C*) (10). This redox-coupled structural change would provide the heme *a* location appropriate for effective electrostatic interaction to the protons on the hydrogen bond network of the H-pathway during the electron transfer through heme *a.*
(5) Ser^382^ in the residues 380-385 in the helix X of subunit I. The redox-coupled structural change in Ser^382^ eliminates one of the water cavities detectable in the water channel connecting the N-side surface entrance of the H-pathway with the hydrogen bond network of the H-pathway (as marked by a blue arrow in Fig. 1) upon complete oxidation of the fully reduced form (Fig. 2*D*). It has been proposed that this structural change blocks back-leaks of protons for pumping on the hydrogen bond network of the H-pathway (10, 13, 14). On the other hand, Rousseau’s group has proposed that the redox-coupled structural changes in the OH group of the hydroxyfarnesyl ethyl group of heme *a*, forming a hydrogen bond to the Ser^382^-OH group upon oxidation, control the proton pumping through the H-pathway (15).

The above five redox-coupled structural changes have been identified by comparison of X-ray structures in the fully reduced and fully-oxidized forms in which all the metal sites are in the reduced and oxidized states, respectively, as described above (4, 8, 9, 10). Thus, it is impossible to identify the metal sites which induce these structural changes. Although the intrinsic redox potentials of the four metal sites are different from each other, selective reduction or oxidation of any single metal site is impossible due to the tight interactions between these metal sites. However, identification of metal sites inducing these structural changes is indispensable for elucidation of the functional roles of these structural changes.

Cyanide, a potent classical respiratory inhibitor, has long been used as an excellent probe of structures and properties of CcO, since 1939 by Keilin and Hartree (16). This reagent has a much stronger affinity to the ferric heme than ferrous heme. This property of cyanide is highly likely to contribute to the identification of the correlation between the metal sites and the redox-coupled structural changes. However, no extensive high-resolution X-ray crystal structural analysis of cyanide-bound CcO under various oxidation states has been reported, though crystal structures of the cyanide-bound fully-oxidized and fully reduced CcOs at 2.0 Å and 2.05 Å resolutions, respectively, have been reported (17, 18). Extensive infrared analyses for the cyanide-bound bovine CcO showed an independent relationship between the low-potential site metals and Fe_*a*3_^3+^ (19, 20). However, infrared identification of the redox-sensitive amino acid residues of CcO has not been reported since experimental conditions for site-specific isotope labeling of amino acid residues of bovine CcO have not been established, although a cell-free protein synthesis system for site-specific incorporation of isotopically labeled amino acids has been reported for a bacterial CcO (21).

Here we report, as a trial for identification of the metal sites which induce these redox-coupled structural changes, the effects of cyanide on these structural changes, examined under various oxidation states by high-resolution X-ray crystallographic analyses. Unexpectedly, the present results showed a clear independent relationship between the structural changes induced by the low-potential sites and those by the O_2_-reduction site. The independence provides a structural basis for the constant proton pump efficiency (H^+^/e^-^ = 1) in each of the four pumping steps and confirms experimentally the proposal that the H-pathway pumps protons. Furthermore, the present cyanide probing revealed a critical role of a transmembrane helix of subunit II for the positive allosteric regulation of CcO function by Higd1a (22), kinetically confirming the function of the O_2_-reduction site as a proton pump element, proposed by the present structural analyses by X-ray crystallography.

## Results

In this paper, the cyanide-bound fully-oxidized, mixed valence (three electron-reduced), and fully reduced (four-electron reduced) forms are abbreviated as CNox, CNmv, and CNred, respectively. In the CNmv, only the Fe_*a*3_ is in the oxidized state (*i.e.*, Cu_A_^1+^, Fe_*a*_^2+^, Cu_B_^1+^, Fe_*a*3_^3+^-CN^-^) (19). Crystals containing CNox, CNmv, and CNred for X-ray diffraction experiments were prepared with the method as described in Experimental procedures and Supporting information (Supporting Text 2 and Fig. S2). Whole procedures of structure determination of the three forms, taking the possibility of the existence of multiple structures into account, are given in Experimental procedures and Supporting information (Supporting Texts 3–5, Table S1–S3 and Figs. S3–S12). The final crystallographic structures (abbreviated as X-ray structures, hereafter) of these three forms are presented in Results and the processes for obtaining them are described in Experimental procedures and Supporting information (Supporting Texts 2–6 and Figs. S2–S16). The X-ray structure of the two azide-bound fully-oxidized form, reported previously (23), showing the structure closely similar to that of the CNred was reexamined for comparison. In this paper, oxygen and nitrogen atoms in the atomic models are colored in red and dark blue, respectively, while carbon atoms are colored differently depending on the oxidation and ligand-binding states.

### The CNox structure (the cyanide-bound fully-oxidized form), essentially identical to that of the fully-oxidized form

**Figure 3:**
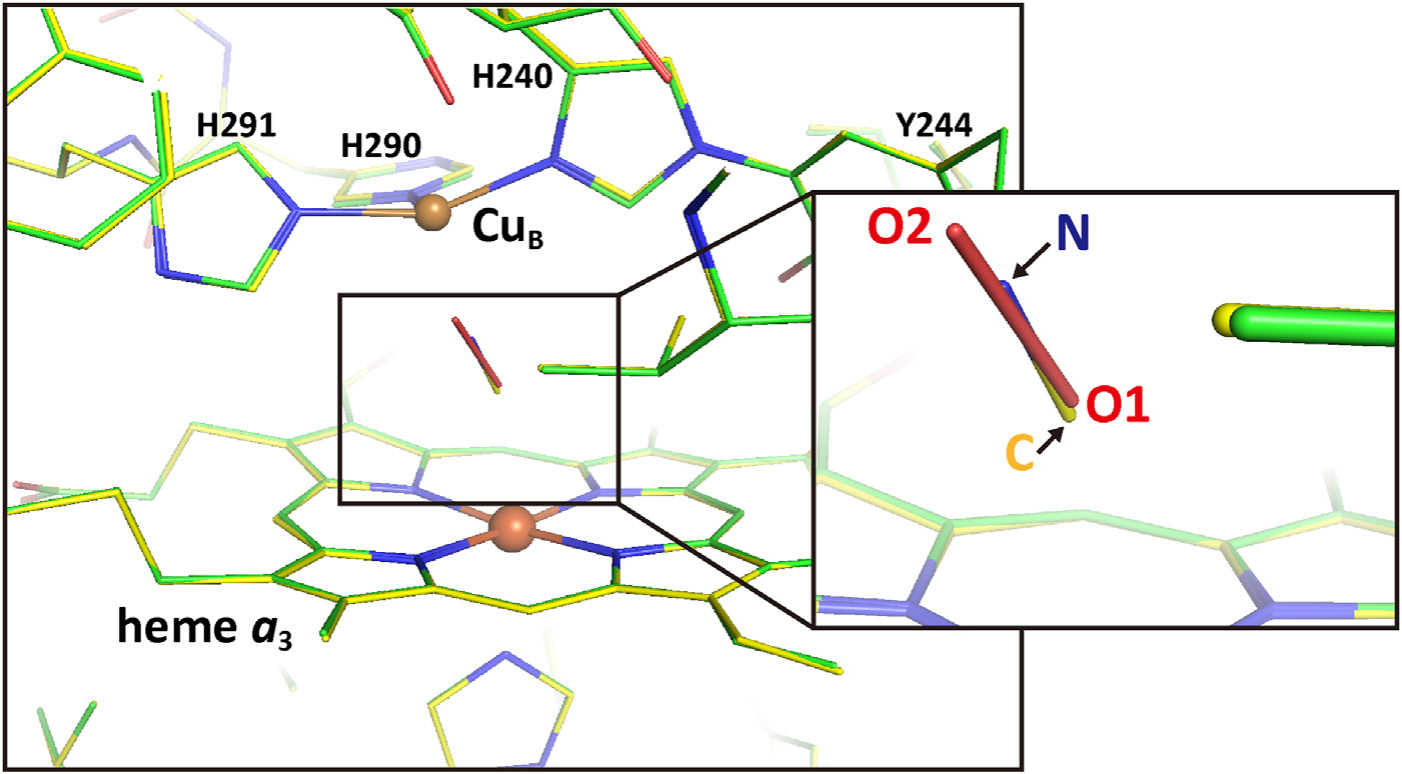
**Structure of the O**_**2**_**reduction site of the X-ray structure of the CNox.** The structure of the O_2_ reduction site of the CNox marked with *yellow* carbon atoms is superimposed with that of the fully-oxidized form (PDBID:5B1A) marked with *green* carbon atoms. The inset shows a magnifying view of the bound ligands.

The X-ray structure of the CNox is illustrated in Figure 3, superimposed on that of the fully-oxidized form colored in green. The inset shows a magnified view of the ligands of both structures, where O1 and O2 denote the oxygen atoms of a peroxide anion ligated to Fe_*a*3_ and Cu_B_, respectively, in the fully-oxidized form. These atomic distances and the superimposed structures (Supporting Texts 6, 1.) indicate that the ligation structures of the CNox are identical to those of the fully-oxidized form except for the bond distances of the CN^-^ and peroxide ligands. No structural difference between the two forms was detectable in the structures other than the O_2_-reduction site (Table 1). The structure detectable in the fully-oxidized form is abbreviated as *ox* in Table 1. Further details in the structural analysis of the CNox are given in Supporting Texts 6, 1.

**Table 1:** Structures of CcO forms at various oxidation and ligand-binding states

Forms	Low-potential sites	O_2_ reduction site	Asp^51^^a^	Arg^438^	Glu^198^	heme *a*^b^	Ser^382^^c^	heme *a*_3_	27–47(II)^d^
Cyanide-bound									
CNox	Fe_*a*_^3+^, Cu_A_^2+^	Fe_*a*3_^3+^-CN^-^-Cu_B_^2+^	*ox*	*ox*	*ox*	*ox*	*ox*	*ox*	*ox*
CNmv	Fe_*a*_^2+^, Cu_A_^1+^	Fe_*a*3_^3+^-CN^-^-Cu_B_^1+^	*red*	*red*	*red*	*ox/red* (28/72)	*ox/red* (58/42)	*CNmv*	*ox*
CNred	Fe_*a*_^2+^, Cu_A_^1+^	Fe_*a*3_^2+^-CN^-^-Cu_B_^1+^-2His^e^	*red*	*red*	*red*	*ox/red* (28/72)	*CNred*	*CNred*	*CNred*
Inhibitor free									
Fully-oxidized	Fe_*a*_^3+^, Cu_A_^2+^	Fe_*a*3_^3+^-O^-^-O^-^-Cu_B_^2+^	*ox*	*ox*	*ox*	*ox*	*ox*	*ox*	*ox*
P/F	Fe_*a*_^3+^, Cu_A_^2+^	Fe_*a*3_^4+^=O^2-^, Cu_B_^2+^-OH^-^	*ox*	*ox*	*ox*	*ox*	*ox*	*ox*	*ox*
O	Fe_*a*_^3+^, Cu_A_^2+^	Fe_*a*3_^3+^-OH^-^, Cu_B_^2+^-OH^-^	*ox*	*ox*	*ox*	*ox*	*ox*	*ox*	*ox*
E	Fe_*a*_^3+^, Cu_A_^2+^	Fe_*a*3_^3+^-OH^-^, Cu_B_^1+^-H_2_O	*ox*	*ox*	*ox*	*ox*	*ox*	*ox*	*ox*
Fully reduced	Fe_*a*_^2+^, Cu_A_^1+^	Fe_*a*3_^2+^, Cu_B_^1+^	*red*	*red*	*red*	*ox/red* (25/75)	*ox/red* (35/65)	*red*	*ox*
N_3_^-^-bound fully-oxidized									
1N_3_^-^-bound	Fe_*a*_^3+^, Cu_A_^2+^	Fe_*a*3_^3+^-N_3_^-^-Cu_B_^2+^	*ox*	*ox*	*ox*	*ox*	*ox*	*ox*	*ox*
2N_3_^-^-bound	Fe_*a*_^3+^, Cu_A_^2+^	Fe_*a*3_^3+^-N_3_^-^, Cu_B_^2+^-N_3_^-^	*ox*	*ox*	*ox*	*ox*	*ox/CNred*^f^ (50/50)	*ox/CNred*^f^ (50/50)	*ox/CNred*^f^ (57/43)

*ox* denotes the structure detectable in the fully-oxidized form. *red* denotes the structure detectable in the fully reduced form but not in the fully-oxidized form. *CNred* denotes the structure detectable in the CNred but undetectable in both the fully-oxidized and fully reduced forms. *CNmv* denotes the location and structure of heme *a*_3_ detectable only in the CNmv. *ox/red* and *ox/CNred* denote multiple structures. The numbers in the parentheses are the percentage of their occupancies, averaged for the two monomers A and B.

a The residues 45-55 of subunit I including Asp^51^.

b The hydroxyfarnesyl ethyl group of heme *a*.

c The residues 377-385 regions of helix X including Ser^382^.

d The residues 27-47 of subunit II.

e Only in this form, only two His imidazole groups are coordinated to Cu_B_.

f The two azide-bound fully-oxidized form (abbreviated as “2N_3_^-^-bound” in the table) induced the structure identical to that of the CNred.

### The CNmv structure (the cyanide-bound three electron-reduced form), showing a unique heme a_3_ structure

**Figure 4:**
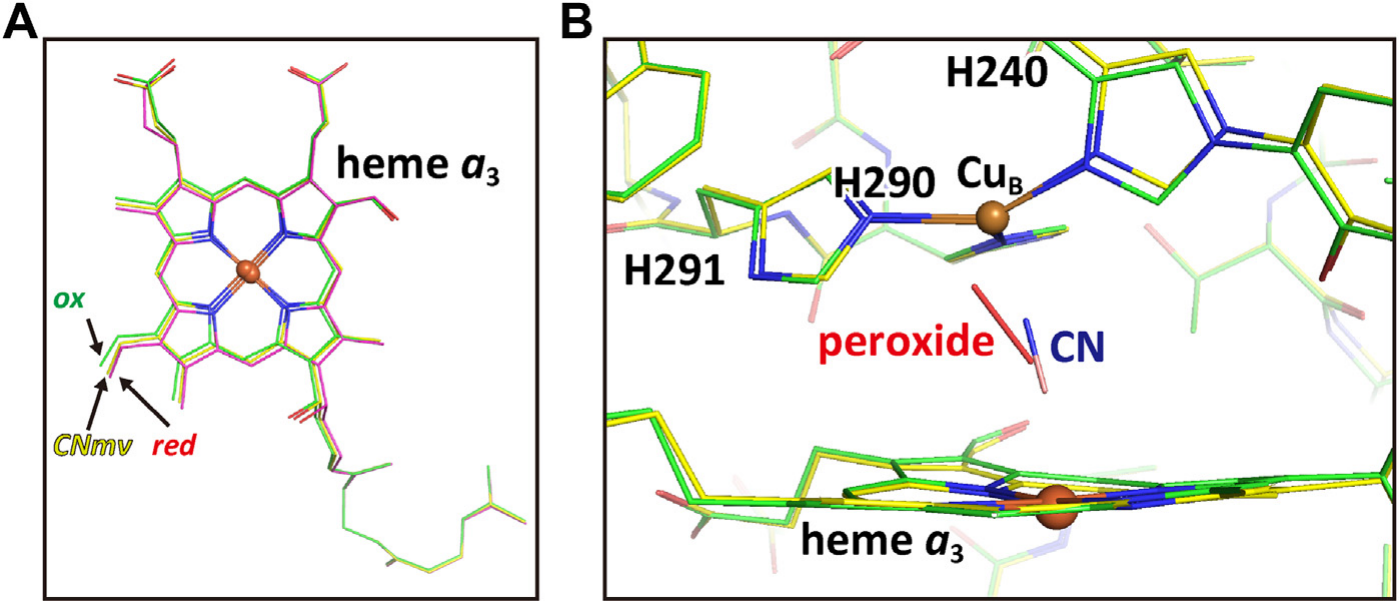
**Structures of heme *a***_**3**_**and CN ligands of the X-ray structure of the CNmv.***A*, heme *a*_3_ structure of the CNmv is marked by *yellow*-colored carbon atoms. The heme *a*_3_ structures of the fully-oxidized form and the fully reduced form with *green*- and *pink*-colored carbon atoms, respectively, are superimposed on that of the CNmv. These three heme planes (labeled with *ox*, *CNmv*, and *red* in the panel, respectively) are on an identical plane. *B*, the X-ray structure of O_2_-reduction site structure of the CNmv marked with *yellow* carbon atoms is superimposed with that of the fully-oxidized form (PDBID:5B1A) marked with *green* carbon atoms.

Heme *a*_3_ in the X-ray structure of the CNmv is illustrated in yellow together with those of the fully-oxidized (green) and fully reduced (red) forms in Figure 4*A*. These three heme planes are on an essentially identical plane, as if the three hemes were translationally shifted with each other. The heme *a*_3_ of the CNmv was located at a position halfway between those of the fully-oxidized (green) and fully reduced forms (red) (Fig. 4*A*). The accuracy of the present X-ray structural analysis, as described in Supporting information (Supporting Texts 6, 2.), is sufficiently high for concluding that heme *a*_3_ of the X-ray structure of the CNmv is assignable as a singular structure and that the structural differences between the three hemes in Figure 4*A* is significant. The heme *a*_3_ structure is designated as *CNmv* in Table 1.

The coordination structure of the CNmv in the O_2_-reduction site is illustrated in yellow and superimposed with the coordination structure of the fully-oxidized form in green in Figure 4*B*. The cyanide ligand of the CNmv coordinated to Fe_*a*3_ in an essentially straight fashion, in contrast to the bent fashion in the CN^-^ ligand of the CNox as described above (Fig. 3). The X-ray structure of the hydroxyfarnesyl ethyl group of heme *a* of the CNmv gave a multiple structure composed of the structures detectable in the fully reduced form and in the fully-oxidized form, as illustrated in Figure 2*C* in a ratio of 72/28 (Table 1). The structure detectable in the fully reduced form is abbreviated as *red* in Table 1. This structure of the hydroxyfarnesyl ethyl group of heme *a* is essentially identical to that of the fully reduced form (ligand free) as described in Table 1. It is noteworthy that even in the fully reduced form, about one-fourth of the hydroxyfarnesyl ethyl group of heme *a* is in the structure identical to that of the fully-oxidized form (*ox* in Table 1). The structure of residues 380–385 in helix X of the CNmv exhibited a multiple structure composed of the structure detectable in the fully-oxidized form (*ox*) and the one detectable in the fully reduced form (*red*) in a ratio of 58/42 (Table 1). The ratio is significantly higher than that for the fully reduced form, 35/65 (Table 1). Asp^51^, Arg^438^, and Glu^198^ did not contain any minor components and were identical to those of the fully reduced form (Table 1).

### The CNred structure (the cyanide-bound fully reduced form), showing that His^290^ at Cu_B_ is replaced with the cyanide at Fe_a3_

**Figure 5:**
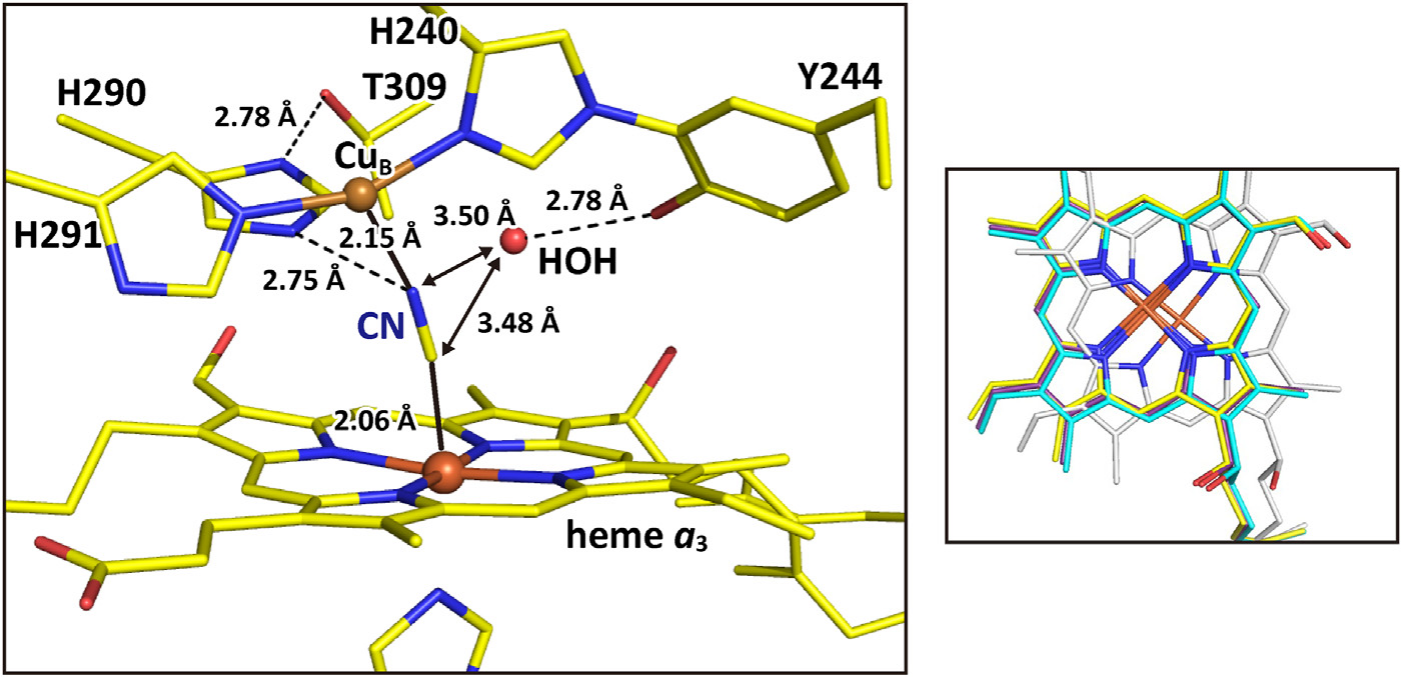
**Structure of the O**_**2**_**-reduction site of the CNred.** The carbon atoms are colored in *yellow*. *Dotted* and *solid* lines show hydrogen bonds and coordination bonds, respectively. The imidazole group of His^290^ is located too far from Cu_B_ (3.16 Å) for coordination. The double arrows indicate the distance between the cyanide ligand and the water molecule hydrogen-bonded to Tyr^244^. The r. m. s. deviation of bond length of the present X-ray structure of the CNred from the ideal structure was 0.018 Å, and the approximate value of SD of atomic position in protein crystal (DPI (40)) was 0.044 Å, as given in Table S2. Inset shows the heme *a*_3_ structures of the fully-oxidized form (*yellow*), CNmv (*beige*), fully reduced form (*blue*), and CNred (*gray*).

The structure of the O_2_-reduction site of the CNred is illustrated in Figure 5. A hydrogen bond with a bond distance of 2.75 Å was detected between N^ε2^ of His^290^ and N of CN^-^. The distance between Cu_B_ and N^ε2^ of His^290^ being 3.16 Å strongly suggests that a coordination bond between Cu_B_ and His290 is broken in the CNred. The distance of 2.15 Å between Cu_B_ and the nitrogen atom of the bound cyanide indicates that His^290^ is replaced with the nitrogen atom of CN^-^ upon the cyanide binding, keeping the hydrogen bond between the His^290^ and OH group of Thr^309^. Any significant structural change is not detectable in the other two His residues (His^240^ and His^291^). A water molecule was hydrogen-bonded to the Tyr^244^-OH group and located 3.48 and 3.50 Å away from the N and C atoms of the bound cyanide, respectively. The water molecule was not detectable in the fully reduced form, though the Tyr-OH groups have enough space for accepting a water molecule. Another remarkable structural change upon formation of the CNred from the CNmv would be the large translational migration of the heme *a*_3_ as revealed in Figure 5 inset. The structure of the heme *a*_3_ in the CNred is designated as *CNred* in Table 1. The details for obtaining this conclusion are given in Supporting information (Supporting Texts 6, 3.). The residues 48-55 of subunit I, Arg^438^, and Glu^198^ of the X-ray structure of the CNred showed those of the fully reduced form (*red* in Table 1). The hydroxyfarnesyl ethyl group of heme *a* of the X-ray structure of the CNred showed the multiple structure closely similar to those of the CNmv and the fully reduced form. These results indicate that the structures of the residues 48-55 of subunit I, Arg^438^, Glu^198^, and heme *a* are shared by the fully reduced form, CNmv and CNred.

**Figure 6:**
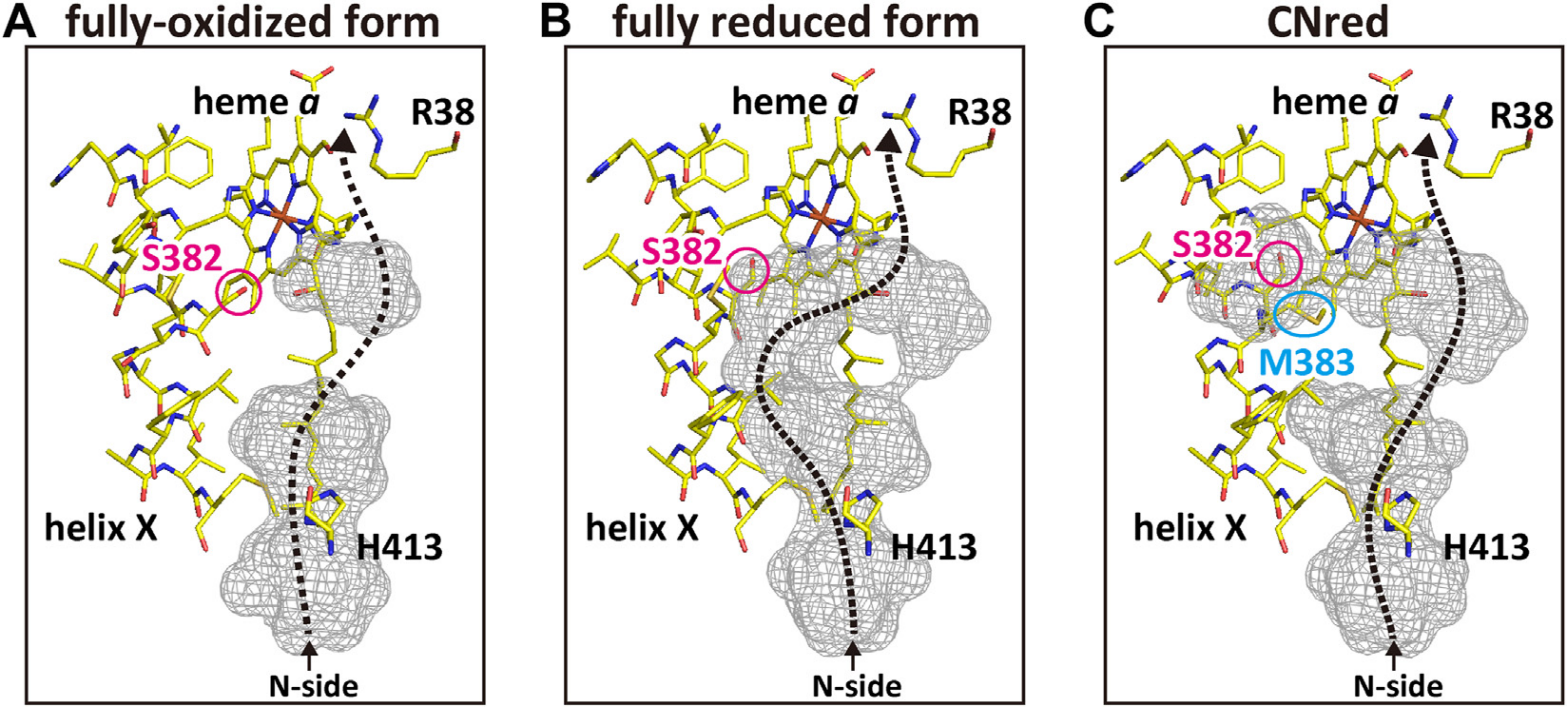
**Water channel structures including the three types of residues 380–390 of subunit I.***A–C*, the fully-oxidized form, fully reduced form, and CNred structures, respectively. The *gray* cages illustrate the water accessible surfaces of cavities in the water channel of the H-pathway, where cavities were calculated by setting radius of 1.20 Å for solvent molecule. The *dotted black arrows* indicate possible water transfer pathways. The carbon atoms in these structures are colored in *yellow*. The *red circles* and *blue oval* label locations of the side chains of Ser^382^ and Met^383^, respectively. The side chains of Met^383^ in panels *A* and *B* are not labeled for the sake of simplicity.

As mentioned in Figure 2*D*, the structural change in the residues Ser^382^ in the residue 380-385 region upon oxidation of the fully reduced form induces blockage of the physiologically relevant water accessibility from the N-side to the Arg^38^ at the N-side end of the hydrogen-bond network of the H-pathway, as illustrated in Figure 6, *A* and *B*. The gray area in the water channel of the H-pathway in the fully reduced form (panel B) denotes the water accessible space composed of several water cavities, each of which can store at least one water molecule. The water cavities enhance water exchange in the water channel, since the movement of the water molecules in the cavities is not restricted with the protein moiety providing the effective proton transfer pathway through the water accessible space as marked by a black dotted arrow. The structural changes in Ser^382^ upon transition to the fully-oxidized form, as marked by red circles in Figure 6, *A* and *B*, indicate that one of the water cavities is eliminated to block the proton transfer through the water cavities in the fully reduced state. An alternative proton transfer pathway through the water channel in the fully-oxidized form is detectable as given in Figure 6*A*. However, it is essentially inactive in the physiological time scale, since water movements in the significant part of the channel is strongly restricted by its narrow space. It has been proposed that this structural change in Ser^382^ blocks back-leakage of the pumping protons from the hydrogen bond network of the H-pathway. It has been shown that the residues 380-385 of the fully reduced form (ligand free) has a multiple structure composed of the fully-oxidized (ox) and fully reduced (red) form structures in a ratio of 35/65 as given in Table 1. The structure of the residues 380-385 in helix X of the X-ray structure of the CNred exhibited a singular structure different from both of the structures detectable in the fully-oxidized and the fully reduced forms. Details in the structural analysis are given in Supporting information (Supporting Texts 6, 3.). The structure (Fig. 6*C*), designated as *CNred* in Table 1, showed that Met^383^ (a blue oval) blocked the main water channel, while the Ser^382^ was located near the site of the residue in the fully reduced form (a red circle). The CNred structure indicates that blockage of the proton back-leak from the hydrogen bond network of the H-pathway is not as tight as in the fully-oxidized form. In fact, the water accessibility remains significant through the second water channel as marked by a dotted arrow.

**Figure 7:**
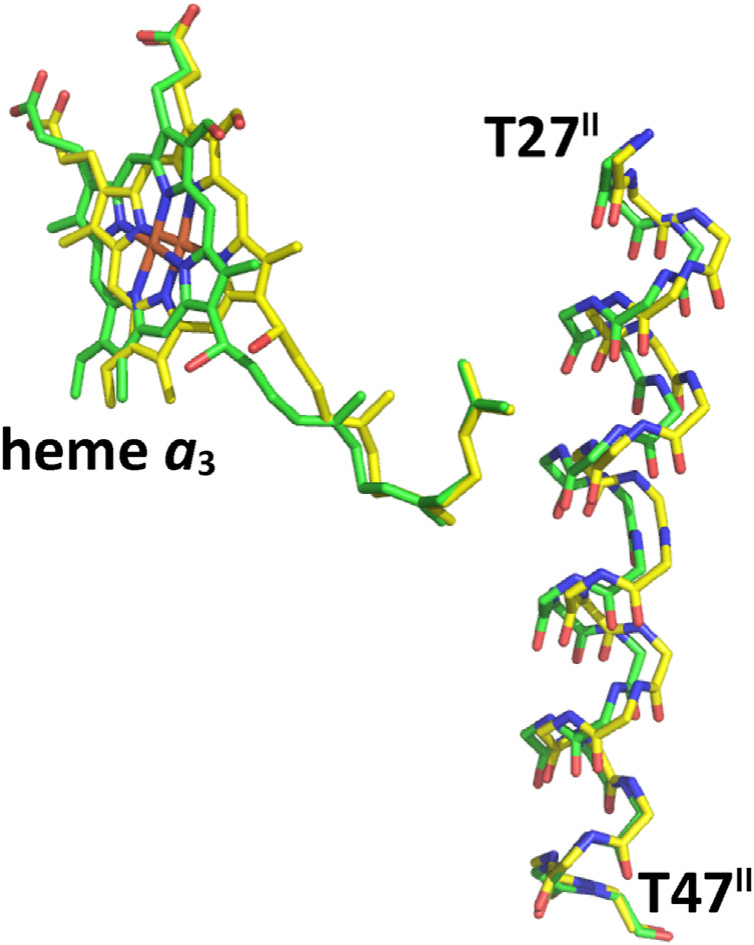
**X-ray structure of residues 27–47 of subunit II of the CNred.** The structure of heme *a*_3_ is included for showing the relative location of residues 27–47 in the transmembrane helix of subunit II. The structure of the fully-oxidized form (*green*) superimposed on that of the CNred (*yellow*).

Figure 7 shows residues 27–47 of subunit II and heme *a*_3_ of the CNred and those of the fully-oxidized form for comparison. The CNmv, CNox, and fully reduced form showed the structure of the residue 27-47 region identical to those of the fully-oxidized from, suggesting that heme *a*_3_ migration upon formation of the CNred, as illustrated in Figure 5 inset, induces the migration of the residues 27-47. As summarized in Table 1, these results indicate that the structures of the residues 48–55 of subunit I, Arg^438^, Glu^198^, and the hydroxyfarnesyl ethyl group of heme *a* of the CNred are identical to those of the fully reduced form and that the residues 380–385 of helix X of subunit I, heme *a*_3_ and the residues 27–47 of subunit II of the CNred have unique structures detectable in the CNred designated as *CNred* in Table 1.

### The structure of the residue 27-47 of subunit II of the two azide-bound fully-oxidized form

It has been reported (23) that the two azide-bound fully-oxidized form showed two different structures in the heme *a*_3_ and the residues 380-385 of helix X in a 1:1 ratio. The two structures are closely similar to those of the fully-oxidized form and the CNred, respectively (Table 1), while a single azide-bound form provides the structures identical to those of the fully-oxidized form except for the ligand binding to the O_2_-reduction site (peroxide *versus* azide) (Table 1). However, no description for the structure of residues 27–47 of subunit II is given in the paper, though higher *B*-factor values are detectable in the region, as given in Fig. S17. Recalculation of the data set given in the previous paper (PDBID: 5Z84) (23) setting multiple structures also in the region showed that residues 27-47 of subunit II of the two azide-bound fully-oxidized form are composed of the two structures essentially identical to those of the fully-oxidized form and CNred. Details for obtaining this conclusion are given in Supporting information (Supporting Texts 6, 4. and Figs. S17 and S18). It is remarkable that the heme *a*_3_ of the CNred, the oxidation and ligand-binding states of which are completely different from those of the two azide-bound fully-oxidized form, provides the structure identical to that of one of the two structures of the two azide-bound fully-oxidized form.

## Discussion

### Relationship between the structural changes induced by the low-potential sites and the O_2_-reduction site

#### The redox sensitive structures when both the low-potential sites are in the reduced state

As summarized in Table 1, the structure of the hydroxyfarnesyl ethyl group of the reduced heme *a* is in an equilibrium state between the fully-oxidized and fully reduced form structures in a ratio of 1:3 within the experimental accuracy in the presence of excess reductant, dithionite, regardless of the oxidation and ligand-binding states of the O_2_-reduction site (*i.e.*, the inhibitor-free fully reduced form (Fe_*a*3_^2+^, Cu_B_^1+^-3His), the CNmv (Fe_*a*3_^3+^-CN^-^-Cu_B_^1+^-3His), and the CNred (Fe_*a*3_^2+^-CN^-^-Cu_B_^1+^-2His)). In the fully-oxidized form, the OH group of the hydroxyfarnesyl ethyl group of heme *a* is hydrogen bonded to Ser^382^ in residues 380–385 of helix X of subunit I (Fig. 2*C*). As given in Table 1, the contents of the fully-oxidized form structure of the Ser^382^ in the fully reduced form and the CNmv are 35% and 58%, respectively, significantly higher than those of the fully-oxidized form structure of the hydroxyfarnesyl ethyl group of heme *a* in these forms (25% and 28%). Thus, a significant amount of Ser^382^ in the fully-oxidized form structure in these forms (10% and 30% for the fully reduced form and the CNmv, respectively) is not hydrogen-bonded to the OH group of the hydroxyfarnesyl ethyl group of heme *a*. In other words, the structure of the OH group of heme *a* is controlled by heme *a*, not by Ser^382^, the structure of which is controlled by the O_2_-reduction site. In the presence of excess reductant, Cu_A_ is also in the reduced state. Although the Cu_A_ site is located significantly distant from the OH of the hydroxyfarnesyl ethyl group of heme *a*, tight interactions between the four metal sites including heme *a* and Cu_A_ have been well known (1, 7). Thus, it is impossible to exclude the possibility that Cu_A_ also participates in controlling of the OH group of the heme *a* structure cooperatively with heme *a*. Asp^51^, Arg^438^, and Glu^198^ are completely in the fully reduced form structure in the presence of excess dithionite, regardless of the oxidation and ligand-binding states of the O_2_-reduction site (the fully reduced form, CNmv and CNred as given in Table 1), indicating that these structures are controlled by the low-potential sites, not by the O_2_-reduction site, as in the case of the structure of hydroxyfarnesyl ethyl group of heme *a.*

#### The redox-sensitive structures when both the low-potential sites are in the oxidized state

In the high resolution X-ray structures of bovine heart CcO in which the low-potential sites are in the oxidized state reported in the previous papers (8, 23) (*i.e.*, P, F, O, E, and azide-bound forms) as well as those in the present paper (the fully-oxidized form and the CNox), all of the structures sensitive to the oxidation states of the low-potential sites (*i.e.*, Asp^51^, Arg^438^, Glu^198^, and the hydoxyfarnesyl ethyl group of heme *a*) are in the fully-oxidized form structure without any minor component, regardless of the oxidation and ligand-binding states of the O_2_-reduction site, as given in Table 1. Thus, the structures sensitive to the low-potential sites in the oxidized state are insensitive to the structural changes in the O_2_-reduction site. That is the case for the structures sensitive to the low-potential sites in the reduced state as described above. These results suggest that the electron transfer process from cytochrome *c* to the O_2_-reduction site *via* the two low-potential metal sites, coupled with the structural changes as described above, is insensitive to the reaction steps in the O_2_-reduction site.

#### The significance of the independent relationship between the structural changes driven by the low-potential sites and those by the O_2_-reduction site

The above finding that the structural changes driven by the low-potential metal sites are insensitive to the reaction stages in the O_2_-reduction site provides a structural basis for the constant pumping proton/electron coupling among the four proton pumping steps in the catalytic cycle as mentioned in Introduction (5). Thus, these structural changes are highly likely to be critically involved in the proton pump. In other words, the present structural findings confirm X-ray-crystallographically the proposal that H-pathway pumps protons. The O_2_-reduction site drives the proton pumping by extracting electrons from the low-potential sites, without perturbing the proton-pump process driven by the low-potential sites. In other words, an identical proton-pump process is repeated four times in each catalytic cycle of CcO.

### Insights into proton-pumping mechanism of CcO obtained by the present cyanide probing

#### Location of heme *a*_3_ controls the water channel structure and the location of the residues 27-47 of subunit II

The present high resolution X-ray structural results summarized in Table 1 indicate that the structure of the water channel (residues 380–385 of helix X including Ser^382^) is critically controlled by the location of the heme *a*_3_ as follows; the heme *a*_3_ located at the fully-oxidized form position (as described in Fig. 4*A*) induces the fully-oxidized form structure for the water channel (Fig. 6*A*) without any minor component. The heme *a*_3_ of CNmv, located halfway between those of the fully-oxidized and fully reduced forms (as illustrated in Fig. 4*A*), induces a multiple structure for the water channel, composed of the fully-oxidized form structure (Fig. 6*A*) the fully reduced form structure (Fig. 6*B*) in equilibrium at 58%/42%, while the heme *a*_3_, located in the fully reduced form (as illustrated in Fig. 4*A*), provides the fully-oxidized form structure and the fully reduced form structure in equilibrium at 35%/65%. The heme *a*_3_ located in the CNred (as illustrated in Fig. 5 inset) gives the water channel of the CNred (Fig. 6*C*) without minor component. The CNred structure of residues 27–47 of subunit II is induced by the CNred structure (location) of heme *a*_3_ as described in Figure 7. The P, F, O, E, and CNox, which show their heme *a*_3_ at the location identical to that of the fully-oxidized form, provide the water channel of the fully-oxidized form. Only these high-resolution X-ray structural analyses are able to identify this critical involvement of the heme *a*_3_ structure (or location) in the structural controls for the proton pump function of CcO. This type of heme *a*_3_ function, (*i.e.*, inducing conformational transitions by its translational movement) has never been mentioned thus far in the field of hemoprotein research in our knowledge.

#### The water channel structures of the CNmv and CNred, suggesting an electrostatic interaction between heme *a* and pumping protons on the H-pathway

It has been shown that binding of two azide ions to the fully-oxidized form induces widening of the water channel of the H-pathway (23) and a significant decrease in the redox potential of heme *a* (24). These results suggest the existence of an electrostatic interaction between heme *a* and pumping protons on the hydrogen-bond network of the H-pathway, since the water channel widening decreases the proton level in the H-pathway above the water channel (23). It has been reported that cyanide binding to Fe_*a*3_^3+^ also significantly decreases the redox potential of heme *a* (25). The present results showed that 42% of the water channel in the CNmv was in the widened state (*i.e.*, in the fully reduced form structure as illustrated in Fig. 6*B*) (Table 1) and the CNred was in an essentially open state as shown in Figure 6*C*. These widened state structures, which appear during the redox titration of the cyanide-bound CcO, are likely to decrease the proton level in the environment of heme *a*. If there are protons interacting with heme *a*, the proton level decrease would weaken the proton affinity of the heme *a*. Thus, the decrease in the redox potential of heme *a* induced by cyanide strongly suggests the existence of an electrostatic interaction between heme *a* and pumping protons on the H-pathway, confirming the proposal based on the structure of the two azide-bound CcO as previously reported (23).

#### Release of His^290^ from Cu_B_ in CNred, suggesting the absence of the proton acceptor near the O_2_-reduction site

In the CNred, one of the three histidine residues, His^290^, is released from Cu_B_ and replaced with a CN^-^-ligand. This histidine imidazole must be released from Cu_B_ by receiving a proton from HCN transferred through the O_2_ pathway through which charged molecules are hard to pass. In fact, pH influence on the cyanide accessibility to the O_2_-reduction site strongly suggests that only protonated cyanide (HCN) is accessible to Fe_*a*3_ (26). However, infrared results indicate that the bound cyanide is in the deprotonated form, CN^-^ (20, 27). That is the case for the azide bound to CcO. Thus, it has long been proposed that the O_2_-reduction site must have a proton acceptor site for the protons released from the protonated ligands, without showing any structural evidence. In the case of the fully-oxidized form, the bound peroxide could receive the proton upon cyanide or azide binding. However, in the fully reduced form, no external ligand is detectable in the O_2_-reduction site (1, 10). Thus, the proton released from the HCN induces the large structural changes on the Cu_B_ site for releasing the His^290^ imidazole to provide the proton-accepting site.

The present result strongly suggests that the O_2_-accessible space including the O_2_-reduction site have neither sufficient number of water molecules for keeping stationary protons released from the externally transported HCN nor any proton-accepting site other than His^290^ in the fully reduced state. That is very likely to be the case in other oxidation and ligand-binding states, since the structure of the protein moiety forming the O_2_ accessible surface including the O_2_-reduction site is insensitive to the oxidation and ligand-binding states in the O_2_-reduction site (4, 8, 10). There are many possible proton-accepting sites other than His^290^ near the O_2_ accessible surface (for example, propionate groups of hemes, Arg^438^, Arg^439^, and Glu^242^). However, no significant structural changes in these possible proton-accepting sites other than His^290^ is detectable upon the cyanide binding, suggesting that these sites, other than His^290^, are tightly sealed from the proton transfer from the O_2_ accessible space. A proton pump mechanism including the D-pathway as the proton pump site, as mentioned in Introduction and Supporting information (Supporting Text 1 and Fig. S1), proposes that Glu^242^ transfers protons from the N-side *via* the D-pathway to a proton-loading site located somewhere near the surface of the O_2_ accessible space and that the loaded protons are pumped to the P-side by another proton transferred by Glu^242^ from the N-side *via* the D-pathway to the O_2_-reduction site for making water molecules from O^2-^ produced by reduction of O_2_. Many mutagenesis results for bacterial CcOs support this proposal (1, 2, 3). However, the most critical structure for this proposal, the proton-loading site, is still yet to be identified. The present results suggesting the absence of the proton-accepting site from the O_2_ accessible space do not support this proposal. Release of His^290^ from Cu_B_^1+^, which is accompanied with the large coordination structural changes as in the case of formation of the CNred, is unlikely to occur during the catalytic cycle. Furthermore, any significant change in the structure (even in its average *B*-factor values) of Glu^242^, which is located at the exit of the D-pathway and exposed to the O_2_ accessible surface, is not detectable among the high resolution structures of bovine heart CcO in various oxidation and ligand-binding states reported thus far (8).

### Possible physiological functions of CcO revealed by the present cyanide probing

#### Allosteric activation of CcO by HigD1a mediated by the residues 27-47 of subunit II confirms the proton pump function of the H-pathway

**Figure 8:**
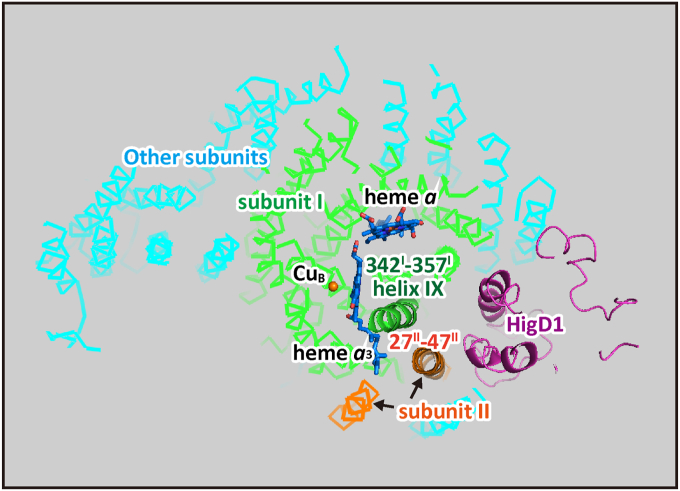
**Possible location of Higd1a helices in CcO.** A possible location of the transmembrane helices of Higd1a, estimated as described previously (22), is shown by *purple*-colored helices. The subunit I helices and two hemes are colored in *green* and *dark blue*, respectively. The two transmembrane helices of subunit II are colored in *yellowish brown*. The helices of residues 27–47 of subunit II and 342–357 of helix IX of subunit I are darkened and labeled by these residue numbers. CcO, cytochrome c oxidase.

Higd1a (hypoxia inducible domain family, member 1A) is a positive regulator of CcO, which is transiently induced under hypoxic conditions and stimulates CcO to improve cell viability under hypoxia (22). Its binding site has been simulated as illustrated in Figure 8 (22), showing that one of the transmembrane helices including the residues 27-47 bridges between heme *a*_3_ and HigD1a. The residues 342–357 of helix IX of subunit I with significantly higher *B*-factor values than those of surrounding residues are also placed between Higd1a and heme *a*_3_. It has been proposed, based on this simulated location of Higd1a, that the bound regulator positively activates heme *a*_3_ function, mediated by these helices (22). The strong interaction between residues 27–47 of subunit II and heme *a*_3_ identified by the present X-ray structural work firmly confirms the above simulation results. It has been shown that the regulator activates CcO allosterically, namely extent of the activation is insensitive to the electron flow rate from cytochrome *c* (22). The strong interaction between the residues 27–47 of subunit II and heme *a*_3_ suggests that heme *a*_3_, activated by HigD1a, enhances allosterically the CcO reaction, indicating that heme *a*_3_ (the O_2_-reduction site) enhances the enzyme activity without perturbing the mechanism of the proton-coupled electron transfer through the low-potential metal sites. On the other hand, the present X-ray structural analysis shows that the redox-coupled structural changes induced by the low-potential metal sites are not influenced by the oxidation and ligand-binding states of heme *a*_3_. Based on the X-ray results, it has been proposed that heme *a*_3_ drives the enzyme reaction without influencing the mechanism of the proton-coupled electron transfer through the low-potential sites. Thus, the allosteric function of heme *a*_3_, revealed by the present X-ray structural analysis for the CNred, kinetically confirms the above X-ray structural proposal that heme *a*_3_ drives the CcO function without perturbing the mechanism of the electron transfer through the low-potential sites.

#### Evolutional conservativity of the residues 27-47 of subunit II

The location and binding characteristic of the residues 27-47 to subunit I is quite well conserved. A subunit of *Thermus thermophilus ba*_3_, corresponding to subunit II of bovine CcO, has only one transmembrane helix, missing the second transmembrane helix corresponding to the one including the residues 27-47 in bovine CcO (28). However, a transmembrane helix, not connected covalently to the *T. thermophilus* subunit II, is located in the subunit I surface at the position essentially identical to that of residues 27–47 of subunit II of bovine CcO. This function of one of the two helices of subunit II has never been mentioned thus far. The finding that the structural changes in residues 27–47 of subunit II are also detectable upon two azide-binding to the fully-oxidized form as described above suggests that the influence of Higd1a to the O2-reduction site is independent to the reaction steps in the O2-reduction site, consistent to the allosteric character of the regulator function.

#### Possible superoxide scavenging function of CcO

Cyanide anion is a model compound of superoxide anion. Thus, the present findings suggest that CcO in the R-form functions as a strong superoxide scavenger. The R-form would readily accept protonated superoxide (HO_2_) to form an O_2_^-^-bound form with protonated His^290^, in the same fashion as the CNred and thus the water channel is widened to decouple the proton-pumping function. The decoupling of the proton-pumping function by widening of the H-pathway would accelerate the electron transfer to the O_2_-reduction site for reduction of the bound superoxide. This scavenging function is likely to be stronger under hypoxia which increases the steady state level of the R-form.

## Experimental procedures

### Intensity data acquisition

Three types of the cyanide-bound CcO crystals (the CNox crystals, the CNmv crystals, and the CNmv/CNred crystal composed of a mixture of the CNmv and CNred) were prepared as described in Supporting information (Supporting Text 2). The asymmetric unit of each of the crystals used in this study contains two monomers of CcO, termed A and B (1, 29). X-ray diffraction experiments for these crystals were performed at a wavelength of 0.9 Å on the BL44XU equipped with an MAR300HE CCD detector at the SPring-8. Crystals were soaked in ethylene glycol as a cryoprotectant for the X-ray experiments at a cryo-temperature to reduce deterioration in crystalline quality. In a representative series of diffraction experiments, the thin edge of a tetragonal plate crystal was aligned parallel to the X-ray beam at a rotation angle of 0.0°. A crystal was shot with X-rays in a helium gas stream at 50 K and translated by 10 μm after each shot to reduce radiation damage. Other experimental conditions for low-resolution data collection were X-ray beam cross-section of 20 μm (vertical) × 20 μm (horizontal) at the crystal, a camera distance of 431 mm, exposure period of 1.0 s, and an oscillation angle of 1.0°. Conditions for high-resolution data collection were an X-ray beam cross-section of 50 μm (vertical) × 30 μm (horizontal) at the crystal, a camera distance of 230 mm, exposure period of 3.0 s, and an oscillation angle of 0.5°. Intensity data processing and scaling were carried out using Denzo and SCALEPACK (30). Structure factor amplitude was calculated using the CCP4 program TRUNCATE (31, 32, 33, 34). Statistics of intensity data collection are given in Table S1.appsec1

### Structure determinations

Initial phase angles of structure factors up to 4.0 Å resolution were calculated by the molecular replacement (MR) method (35) using the structure of the fully-oxidized form, previously determined at 1.5 Å resolution (PDBID: 5B1A). The phases at 5.0 Å resolution were extended to the highest resolution of each data set by density modification (DM) (36) coupled with noncrystallographic symmetry (37, 38) averaging using the CCP4 program DM (39). The resultant phase angles (α_MR/DM_) were used to calculate the electron-density map (MR/DM map) with Fourier coefficients |*F*o|exp(*i*α_MR/DM_), where |*F*o| is the observed structure factor amplitude. Further structural determination procedures consisted of the following three steps, (1) structural determination of a model with a singular structure, (2) multiple structure identification, and (3) determination of the structure of the O_2_-reduction site and final structural refinements, as in the previous paper (8). Details in these steps are described in Supporting Texts 3-5. Statistics of the final stage of the refinements are given in Table S2. A summary of the present X-ray structural determination is given in Table S3.

## Data availability

The atomic parameters and structure factors (PDB ID: 7VUW, 7VVR, and 7W3E, for CNox crystal, CNmv crystal, and CNmv/CNred crystal) have been deposited in the Protein Data Bank (http://wwpdb.org/). All the other data are contained within this manuscript.

## Supporting information

This article contains supporting information including Figs. S1–S18, Supporting Text 1 to 6, Tables S1–S3, and references (41, 42, 43, 44, 45, 46, 47, 48, 49, 50, 51, 52, 53, 54, 55, 56, 57, 58, 59, 60, 61, 62, 63).

## Conflict of interest

The authors declare that they have no conflicts of interest with the contents of this article.
