# Supplementary material for: Crystallographic cyanide-probing for cytochrome c oxidase reveals structural bases suggesting that a putative proton transfer H-pathway pumps protons
Source: J Biol Chem. 2023 Sep 22;299(11):105277. doi: 10.1016/j.jbc.2023.105277 (PMC10598403; doi:10.1016/j.jbc.2023.105277)
Supplement: Supporting information [file mmc1.pdf]

Supporting Information

Crystallographic cyanide-probing for cytochrome *c* oxidase reveals structural bases suggesting that a putative proton transfer H-pathway pumps protons

Atsuhiro Shimada, Jumpei Baba, Shuhei Nagao, Kyoko Shinzawa-Itoh, Eiki Yamashita, Kazumasa Muramoto, Tomitake Tsukihara, and Shinya Yoshikawa[†]

The supporting information includes:

1. Figures S1–S18
2. Supporting Texts 1-6
3. Tables S1-S3
4. Supporting References (41-63)

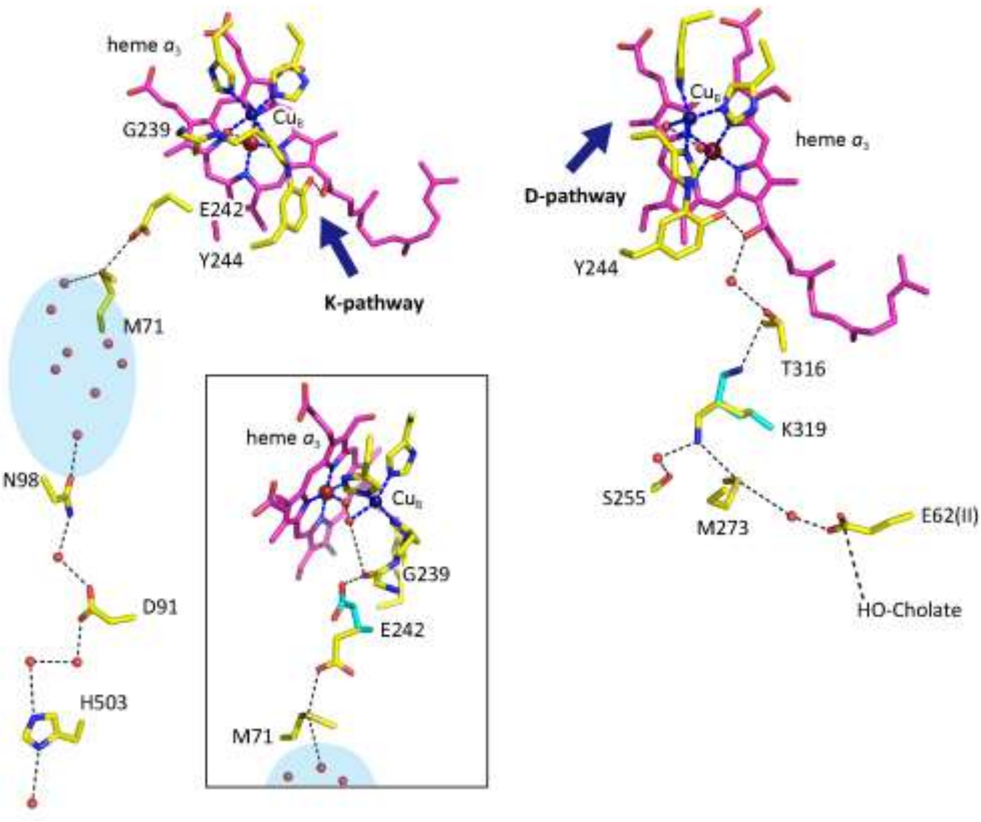

Figure S1. Atomic models of the D- and K-pathways. Purple models denote heme a_3. The dark blue, red and beige portions of amino acids are nitrogen, oxygen and sulfur atoms, respectively. Dotted and broken lines are hydrogen and coordination bonds, respectively. The dark blue spheres and the large red spheres are the Cu_B and Fe_{a3} atoms, respectively. The two metal ions form the O_2-reduction site. The D- and K-pathways are shown in the left and right illustrations, respectively. The junction points for the K- and D-pathways are denoted by blue arrows. The inset in the left illustration, shows a possible hydrogen-bond network from Glu^{242} to the O_2-reduction site. The light-blue oval indicates a water cluster, including fixed waters marked by red small spheres. The blue structures denote possible structural changes for transferring protons. His^{503}, Asp^{91} and Glu^{62} are located at or near the N-side surface of CcO. The O_2-reduction site receives protons for making waters from the N-side through the D and K pathways and electrons from heme *a* (not shown for the sake of simplicity). Reprinted with permission from Reference (1). Copyright 2015 American Chemical Society.

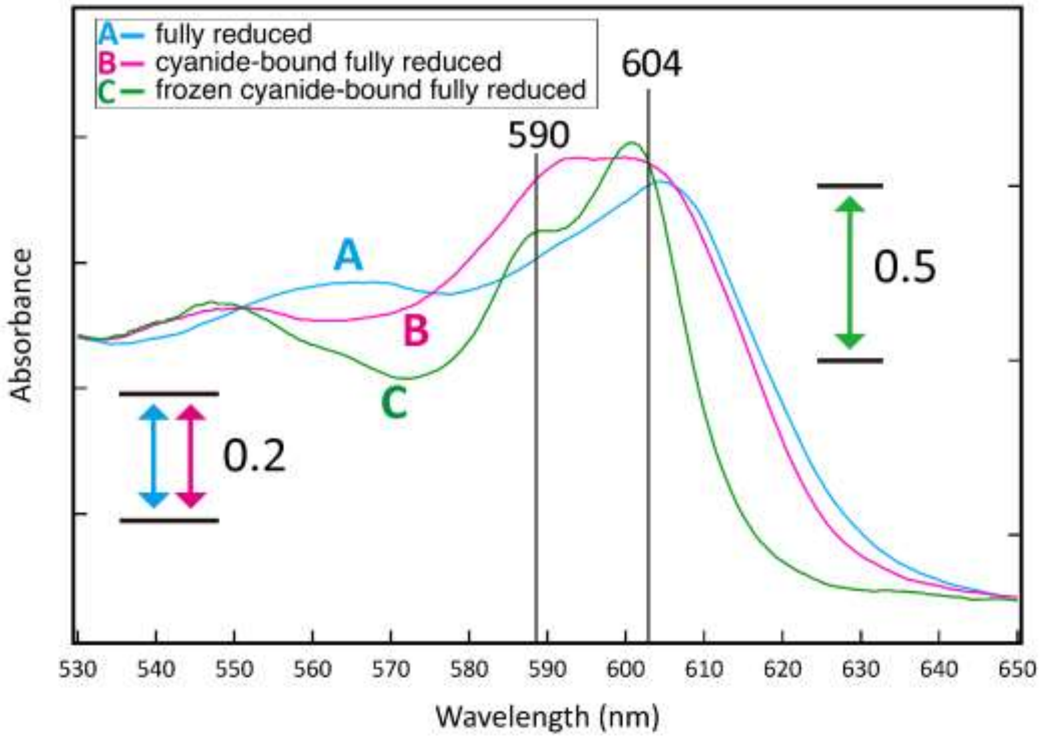

Figure S2 Absorbance spectra of the CNred and CNmv/CNred crystals. *A, B,* absolute absorbance spectra of the fully reduced form (A in cyan) and CNred (B in purple) crystals placed in a hollow slide for light microscope tightly sealed with a cover glass without air space. The spectra were taken in a custom-made absorbance spectrophotometer at 4 ˚C, placing the crystal in the shape of a tetragonal plate perpendicular to the micro light beam. Crystals with a closely similar size were used for the absorbance measurement. *C*, an absorbance spectrum of a CNmv/CNred crystal (colored in green), frozen in a small loop for X-ray diffraction experiment. Because of the crystal size and the angle against the light beam, the effective light path in the crystal in the loop is longer than that for the measurement in the hollow slide giving the spectra A and B and thus higher absorbance were obtained.

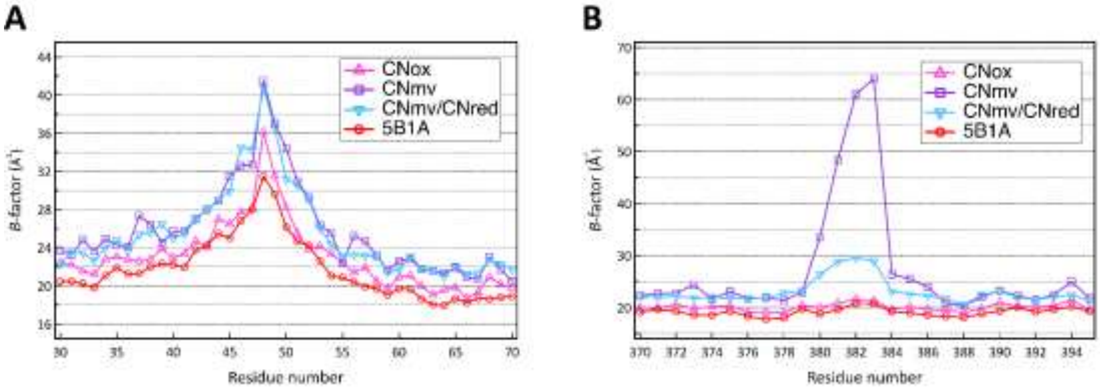

Figure S3 Distributions of *B*-factors of averaged for main chain atoms of each residue in the data sets of the fully-oxidized form (PDBID: 5B1A), CNox, CNmv, and CNmv/CNred crystals. *A*, for residues 30–70 of subunit I. *B*, for residues 370–395 of subunit I**.**

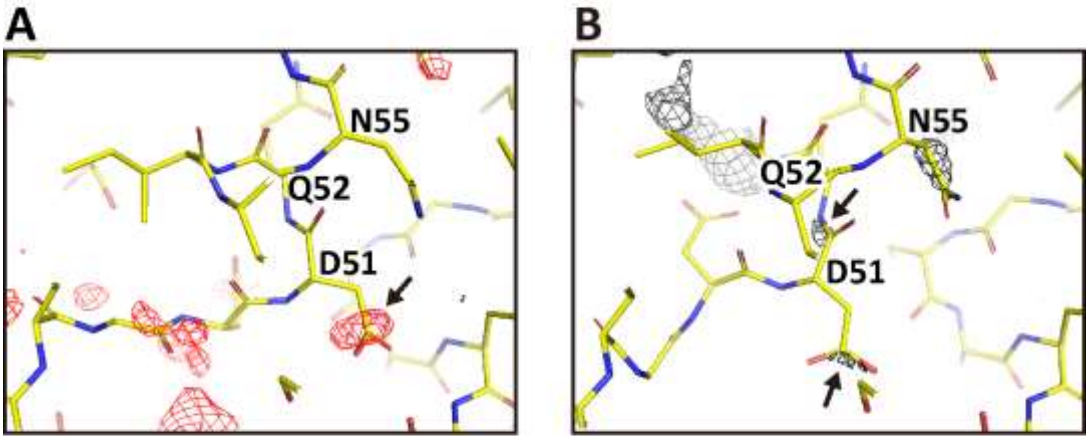

Figure S4. ***Fo-Fc*** **map of residues 48–55 of subunit I of the CNox data set, drawn at 3.0** σ. *A,* negative peaks (red cages) on the atomic model of the fully-oxidized form structure. *B*, positive peaks (black cages) on the atomic model of the fully reduced form structure. Arrows label the peaks which appear at the atom positions of the atomic models.

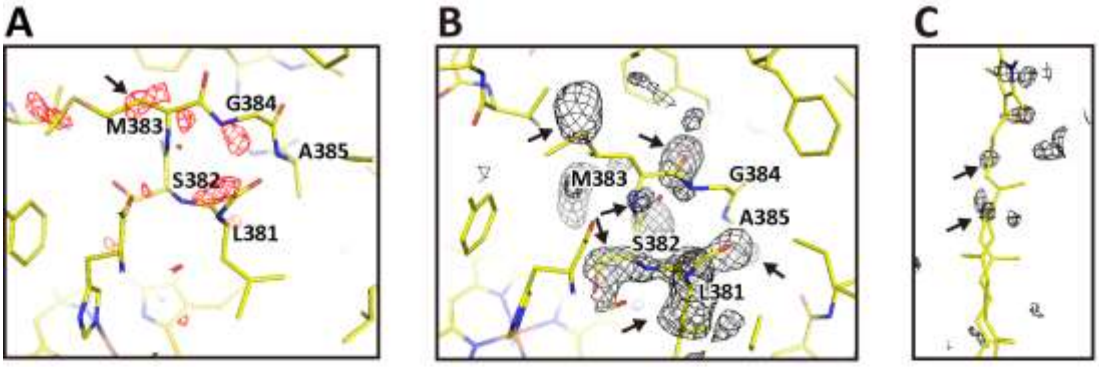

Figure S5. ***Fo-Fc*** **map of the CNmv data set.** *A*, negative peaks (red cages) in residues 380–385 of subunit I, drawn at 3.04 σ, on the atomic model of the fully-oxidized form structure. *B*, positive peaks (black cages) in residues 380–385 of subunit I, drawn at 3.04 σ, on the atomic model of the fully reduced form structure. *C*, positive peaks (black cages) in the hydroxyfarnesyl ethyl group of heme *a,* drawn at 3.0 σ on the atomic model of the fully-oxidized form structure. Arrows label the peaks which appear at the atom positions of the atomic models.

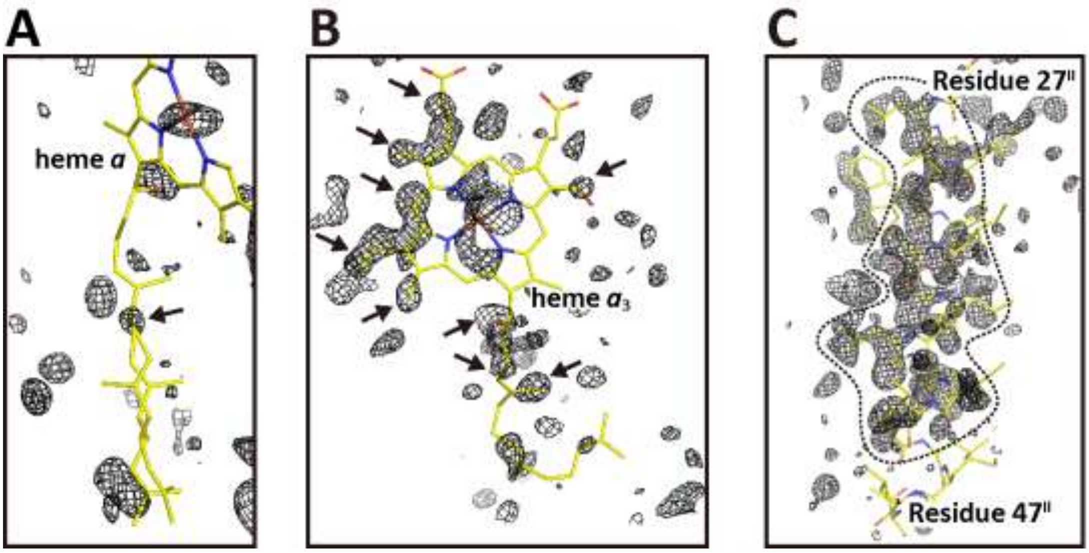

Figure S6. *Fo-Fc* map of the CNmv/CNred data set, drawn at 3.0 σ. *A*, positive peaks (black cages) in the hydroxyfarnesyl ethyl group of heme *a*, on the atomic model of the fully-oxidized form structure. *B*, positive peaks in heme a_3 structure region on the atomic model of the CNmv structure. C, positive peaks (black cages) in residues 27–47 of subunit II on the atomic model of the CNmv data set which is identical to the fully-oxidized form structure in this region. Arrows in panels *A* and *B* indicate the peaks which appear at the atoms of the atomic models. In panel C, most of the positive peaks inside the dotted loop are located on the atomic model of CNmv data set.

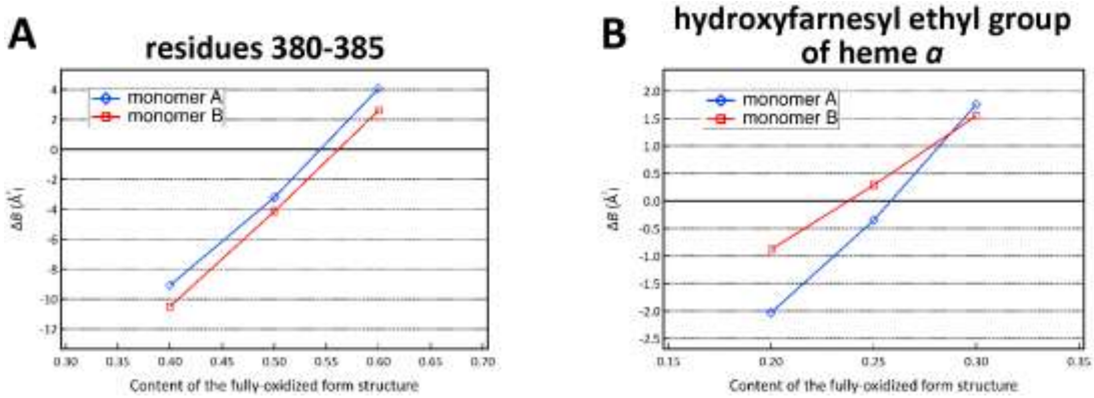

Figure S7. **Δ*B*-plot analyses for the CNmv data set.** Δ*B* values, defined as (average *B*-factor value of the refined fully-oxidized form structure) – (average *B*-factor value of the refined fully reduced form structure), were determined at various content of the fully-oxidized form structure and plotted against the content of the fully-oxidized form structure. *A*, for residues 380–385 of subunit I. *B*, for the hydroxyfarnesyl ethyl group of heme *a*. The blue and red plots are those of monomers A and B, respectively.

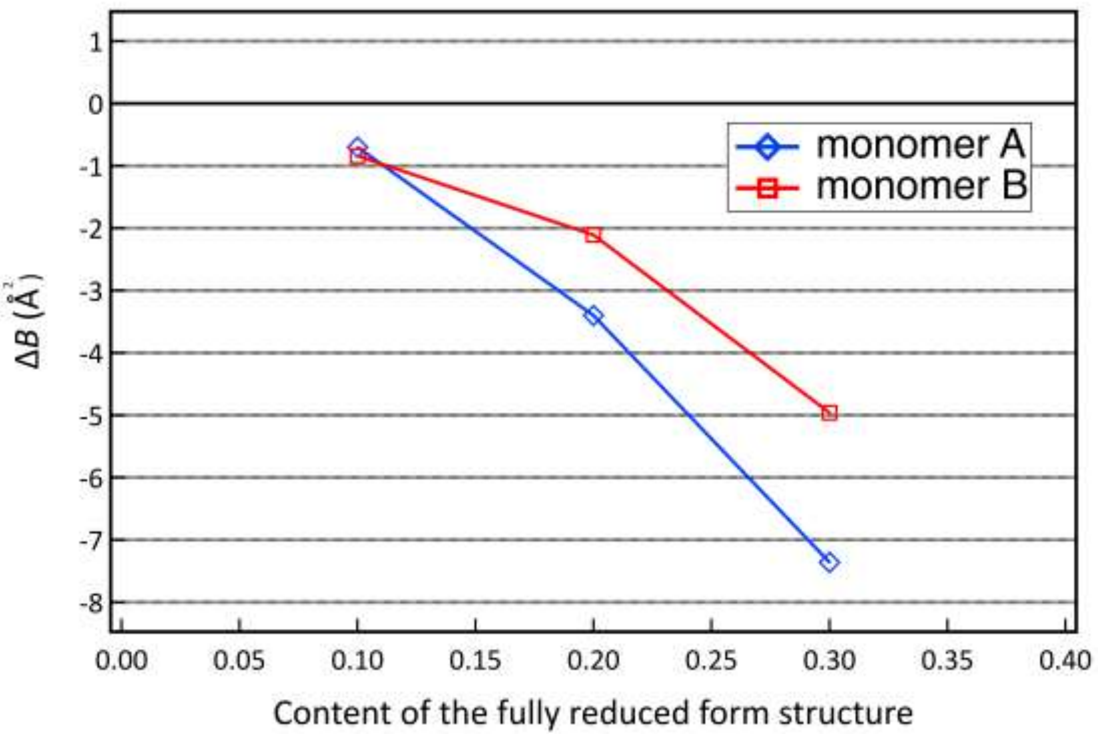

Figure S8. **Δ*B*-plot analyses for the CNox data set.** The Δ*B* values, defined as described in the legend of Fig. S7, were determined for the fully-oxidized and fully reduced form structures in residues 48–55 of subunit I and plotted against the content of the fully reduced form structure for the monomers A (blue plot) and B (red plot).

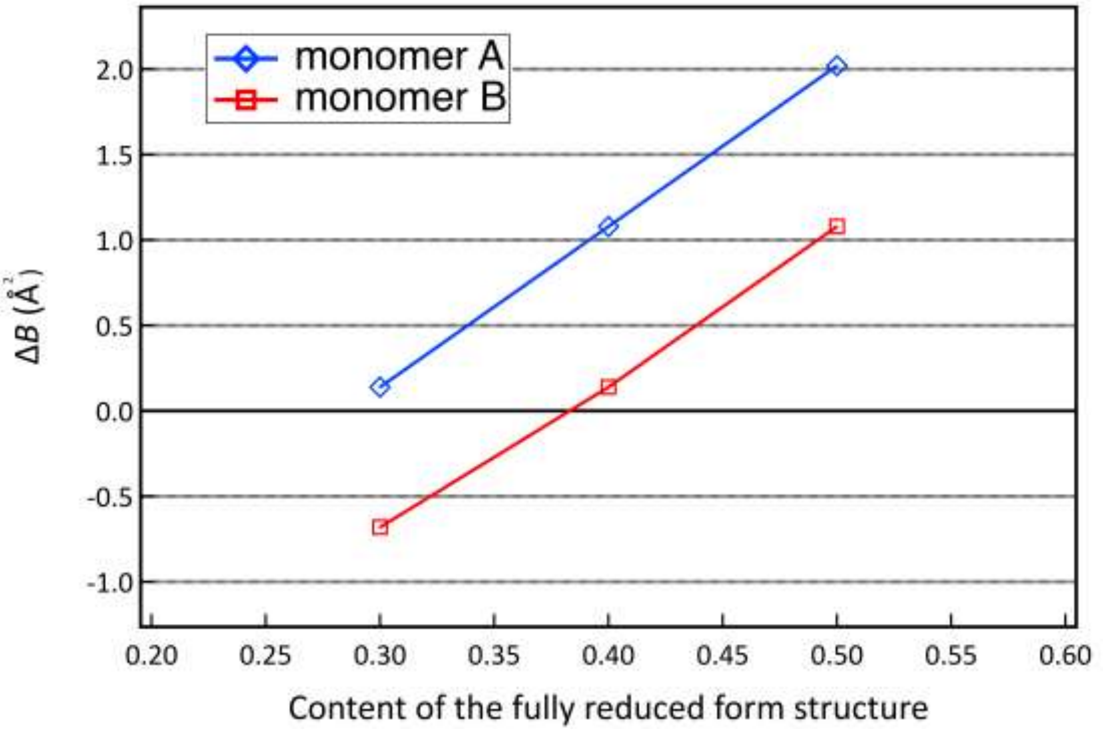

Figure S9. **Δ*B*-plot analyses for the residues 380-384 region of subunit I of the CNmv/CNred data set.** Δ*B* values, defined as (average B-factor value of the refined fully reduced form structure) – (average B-factor value of the refined CNred structure), were determined for the fully reduced form structure and CNred structure included in the residues 380–384 region of subunit I of CNmv/CNred data set and plotted against the content of the fully reduced form structure for the monomers A (blue plot) and B (red plot).

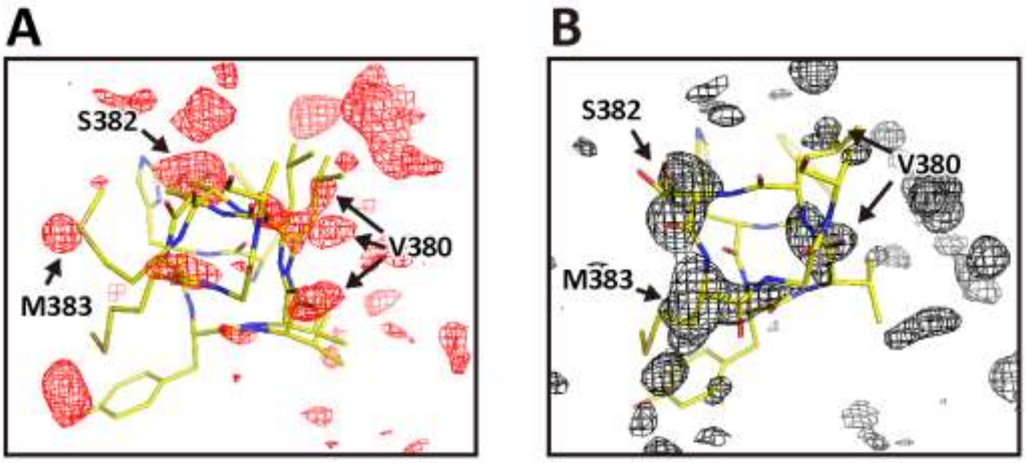

Figure S10. ***Fo-Fc*** **maps of residues 380–385 of subunit I of the CNmv/CNred data set with the multiple structure consisting of the fully reduced form structure and CNred structure.** The content ratio of the two components (the fully reduced form structure and CNred structure) was determined by the Δ*B*-plot analysis given in Fig. S9**.** The maps are drawn at 3.0 σ. *A*, negative peaks on the atomic models of the fully reduced form structure and the CNred structure. *B*, positive peaks on the atomic model of the fully-oxidized form structure. Arrows in panels *A* and *B* indicate the peaks which appear at the atoms of the atomic models.

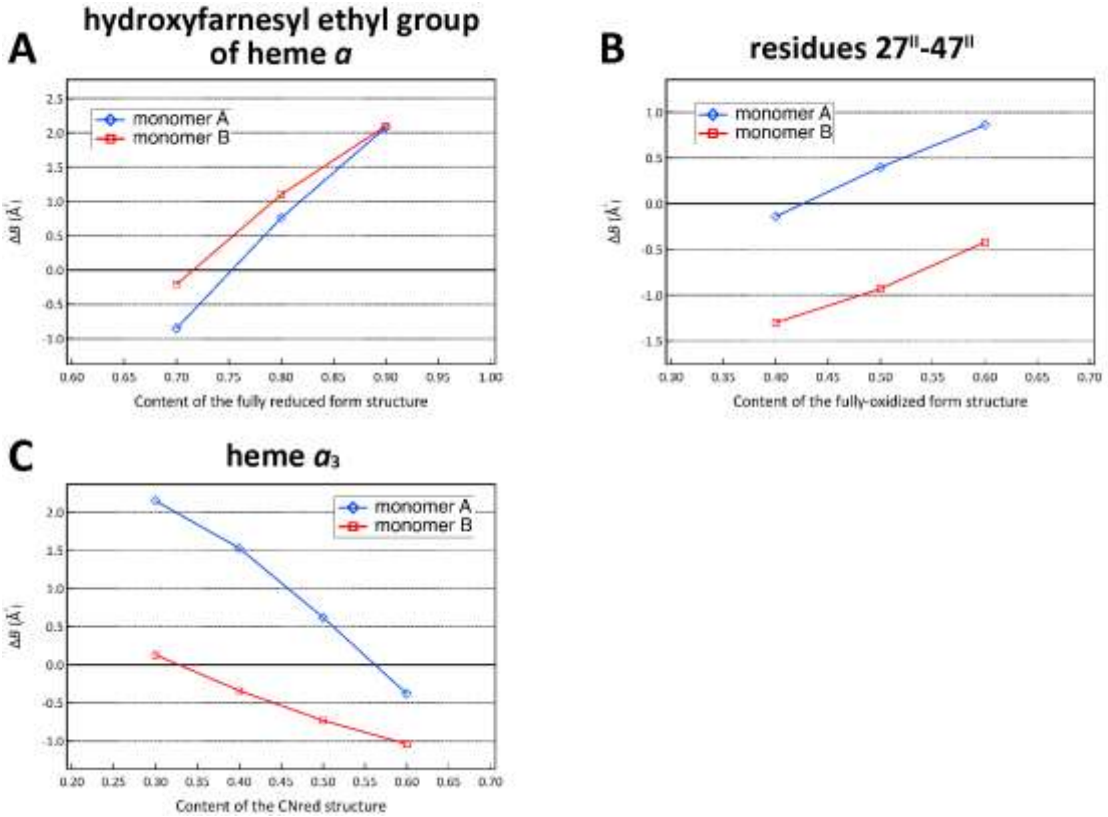

Figure S11. **Δ*B*-plot analyses for the regions of the CNmv/CNred data set other than the residues 380– 385 of subunit I.** *A*, for the oxidized-form structure and the fully reduced form structure in the hydroxyfarnesyl ethyl group of heme *a*, Δ*B*, defined as (average *B*-factor value of the refined fully reduced form structure) – (average *B*-factor value of the refined fully-oxidized form structure), were plotted against the content of the fully reduced form structure. *B*, for the fully-oxidized form structure and the CNred structure in residues 27–47 of subunit II, Δ*B* values, defined as (average *B*-factor value of the refined fully-oxidized form structure) – (average *B*-factor value of the refined CNred structure), were plotted against the content of the fully-oxidized form structure. *C*, for the CNmv structure and the CNred structure in heme a_3, Δ*B* values, defined as (average *B*-factor value of the refined CNmv structure) – (average *B*-factor value of the refined CNred structure), were plotted against the content of the CNred structure. The blue and red plots are those of monomers A and B, respectively.

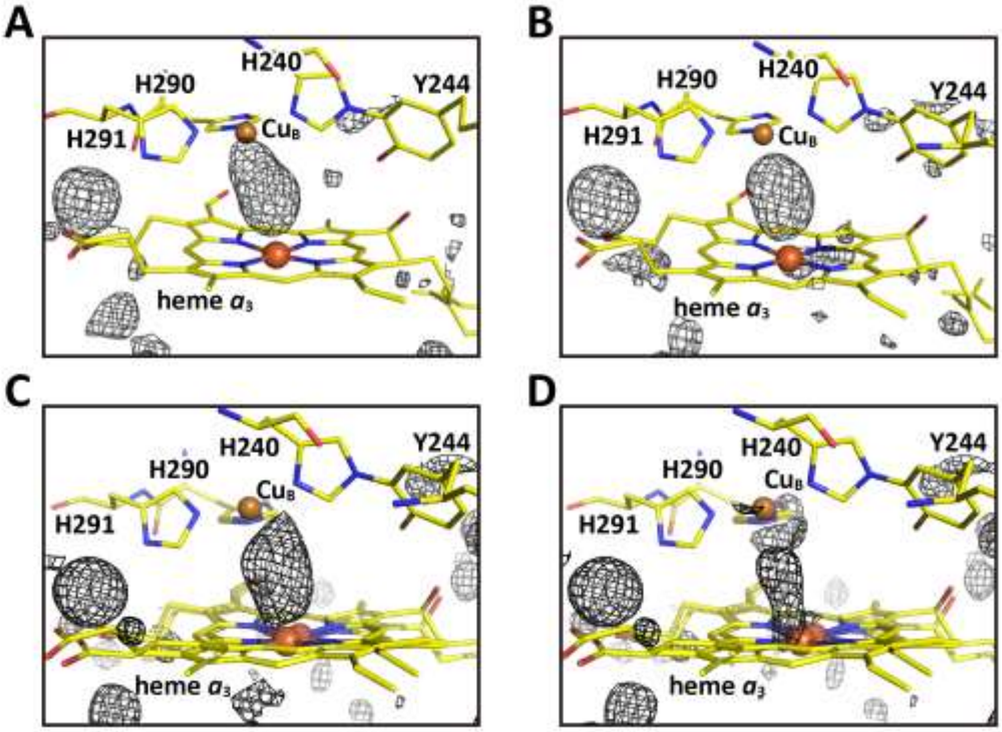

Figure S12. ***F*o-*F*c maps of the O_2 reduction sites of the CNox, CNmv, and CNmv/CNred data sets**. Refinement was performed on a structure in the absence of any ligand in the O_2 reduction site and without the water molecule bridging the two propionate groups of heme a_3 and the *F*o-*F*c map was calculated. *A, B,* and *C*, calculated maps for the CNox, CNmv, and CNmv/CNred data sets, respectively. The electron density cages were drawn at 3.0 σ. Structures of protein moiety and hemes are drawn as stick models, and the Fe atoms in heme a_3 and Cu atoms in the Cu_B site are indicated by brown spheres. Oxygen, nitrogen, and carbon atoms in the atomic models are shown in red, dark blue, and yellow sticks, respectively. *D*, *F*o-*F*c map, calculated for the structure of the CNmv/CNred data set, locating CN^- ligand with 50% occupancy at the CNred heme a_3 site. The electron density cages were drawn at 3.0 σ.

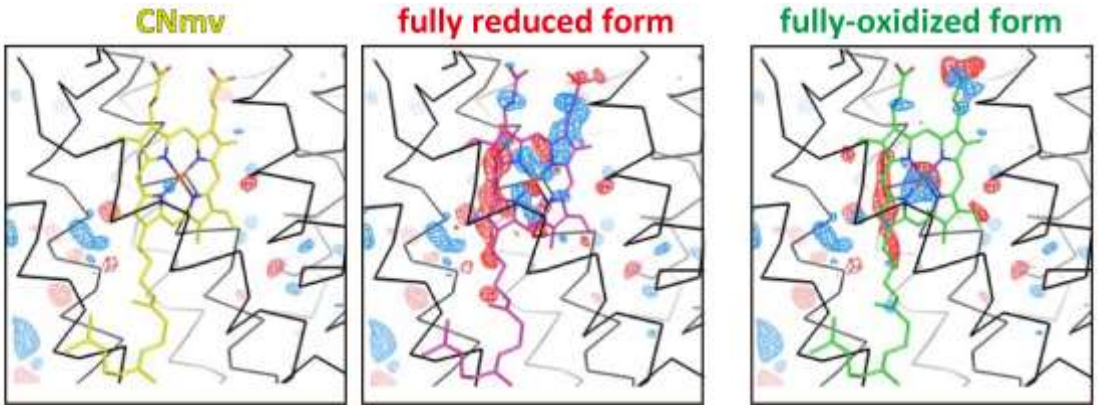

Figure S13. ***Fo-Fc*** **maps of X-ray structures of CNmv, each with a different heme** $\boldsymbol{a_3}$ **structure.** The left, center, and right panels show those with hemes a_3 structures of the CNmv, the fully reduced form, and the fully-oxidized form, respectively. Residual densities of *Fo-Fc* maps (positive and negative in blue and red, respectively) are drawn at 4.0 σ level. The map of CNmv structure (left panel) shows residual densities significantly lower than those of the other two models. These maps indicate that the present X-ray crystal structure analysis can distinguish the heme a_3 of the CNmv from those of the fully reduced and fully-oxidized forms.

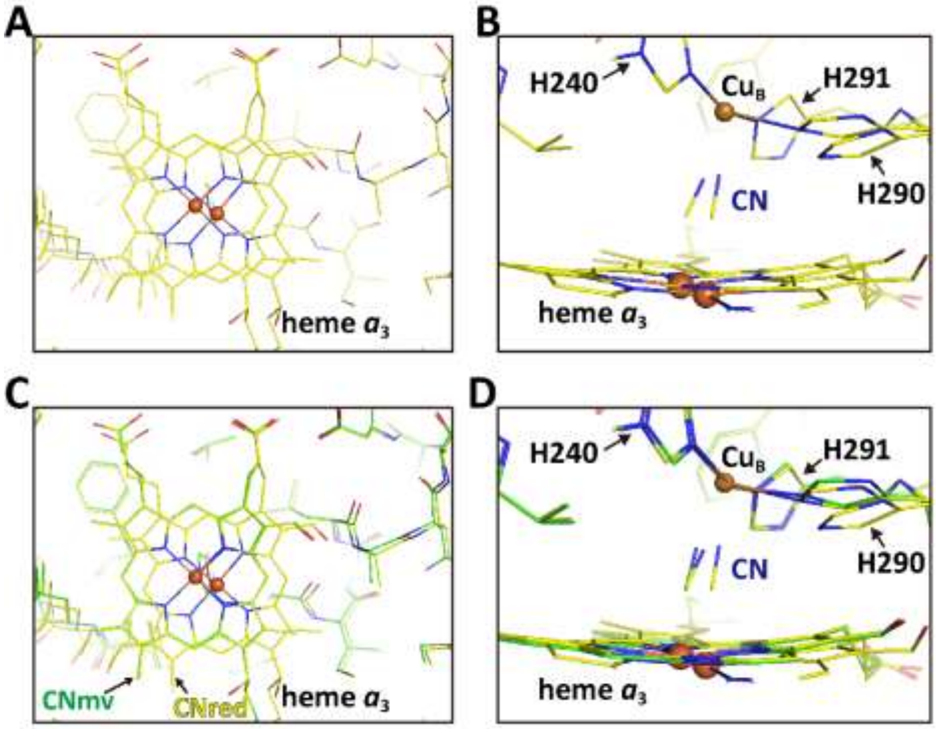

Figures S14. The structures of the heme a_3 and the cyanide-ligand of the CNmv/CNred data set. Nitrogen and oxygen atoms are colored in dark blue and red. Carbon atoms in the structures of the CNmv/CNred and CNmv data sets are in yellow and green, respectively. *A*, structure of heme a_3, *B*, the ligand-binding structure of the O_2 reduction site, *C* and *D*, the structures of heme a_3 and the ligand binding structure of the O_2 reduction site of the CNmv data set with the carbon atoms colored in green are superimposed on the structures of the CNmv/CNred data sets with yellow carbon atoms given in panel A and B of this figure, respectively.

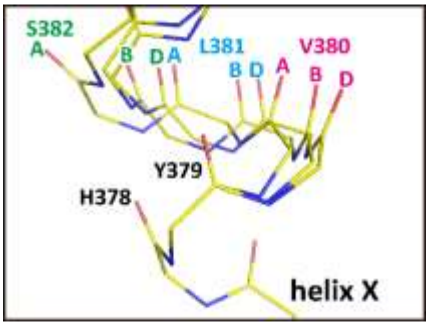

Figure S15. The final structures of three components of the peptide back born of residues 377–383 of subunit I of the CNmv/CNred data set. The relative locations of the peptide back bones of three structures, designated as A, B, and D, detectable in the structure of the CNmv/CNred data set are indicated by alphabetical labeling on the peptide C=O groups of residues 380, 381, and 382. The structures, A, B, and D, are assignable to those of the fully-oxidized, fully reduced and CNred forms, respectively.

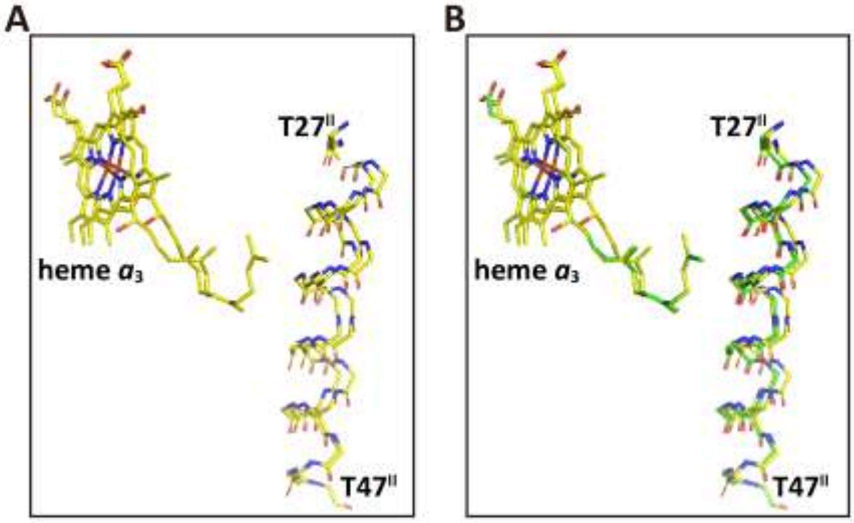

Figure S16. Structures of residues 27–47 of subunit II of the CNmv/CNred data set. The structure of heme a_3 is included for showing the relative location of residues 27–47 in the transmembrane helix of subunit II. *A*, the multiple structures in residues 27–47 and heme a_3. *B*, the structure of the CNmv data set (green) superimposed on that of the CNmv/CNred data set (yellow) in panel A.

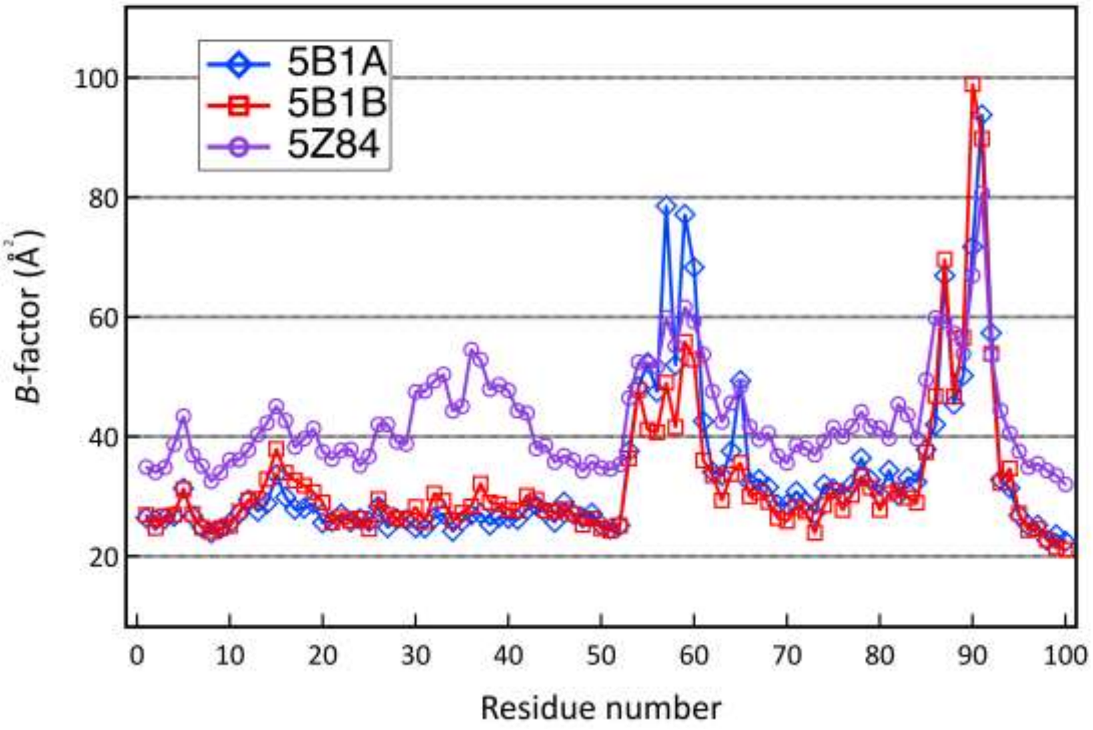

Figure S17. ***B*-factor values averaged for main chain atoms of each residue of subunit II.** The blue, red and purple points denote the *B*-factor values of the fully-oxidized (PDBID: 5B1A), fully reduced(PDBID:5B1B) and two azide-bound fully-oxidized(PDBID:5Z84) forms, respectively

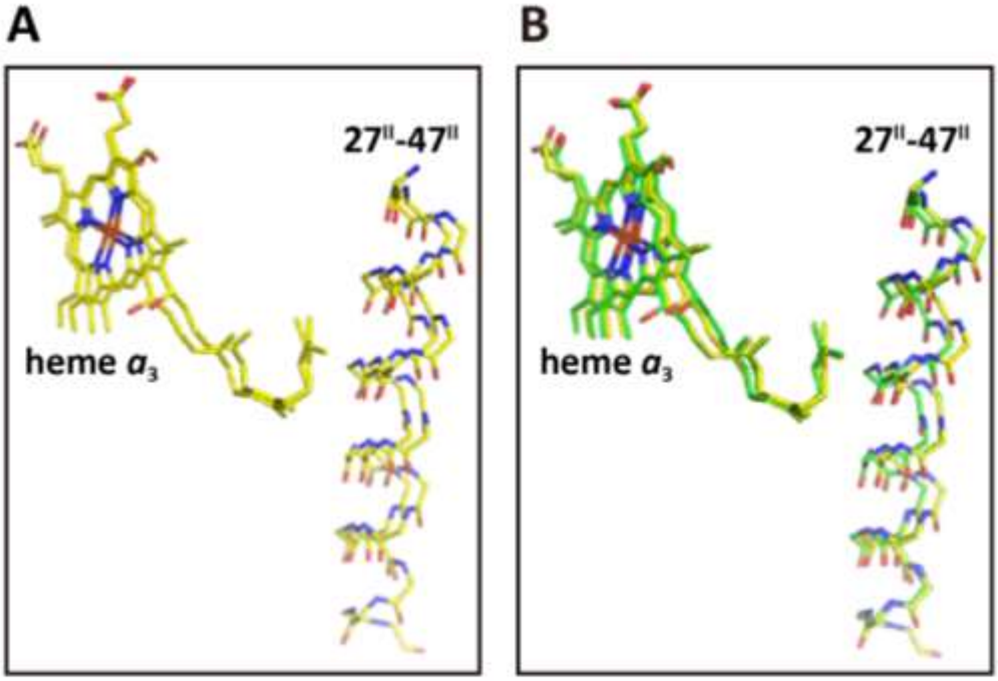

Figure S18. Structure of residues 27–47 region of subunit II of the two azide-bound fully-oxidized form. *A*, the structure of the residues in residues 27–47 of subunit II of the two azide-bound fully-oxidized form, obtained by recalculation of the data set (PDBID:5Z84) reported (23), including the heme a_3 structure for comparison. *B*, the structure of the CNmv/CNred data set with carbon atoms in green superimposes on the structure given in panel A with yellow carbon atoms.

Supporting Text 1.

A summary of the discussions on the proton pump mechanism of cytochrome c oxidase.

1. D-pathway mechanism.

It has been proposed that D-pathway transfers protons both for pumping (pump proton) and for making water from the fully reduced dioxygen (O^{2-}) (chemical proton), based on the mutagenesis results for bacterial CcOs, as follows: A D-pathway mutation for bacterial CcOs, Glu^{242}Gln (in this article, numbering for bovine CcO is used unless otherwise noted.) abolishes completely the enzyme activity by blocking the P→F and F→O transitions (1, 2), while an another D-pathway mutation, Asn^{98}Asp, decouples proton pump function from the electron transfer for the O_2-reduction, namely, the mutation inhibits the proton pump without influencing its electron transfer activity (41). This proposal is designated as D-pathway mechanism in this article. It is noteworthy that the above proposal that D-pathway transfers both pump and chemical protons is one of the simplest interpretations for the above mutation results. In other words, it is impossible to identify the location of the site for a function only by a mutation of the candidate site. For example, although Asn^{98}Asp mutation abolishes the proton pumping function, it is impossible to exclude the possibility that the mutation induces a structural change in a proton conducting pathway for pumping protons other than the D-pathway. In fact, it is not clear why Asp^{98} in the D-pathway blocks the proton transfer through the D-pathway (3). As described above, D-pathway transfers chemical protons to the O_2-reduction site. Thus, an effective mechanism for preventing any spontaneous transfer of the pumping protons to the O_2-reduction site is a prerequisite for the pumping protons to be transferred through the D-pathway without dissipation of the pumping energy.

As described in Supporting Figure S1A, X-ray structures of bacterial and bovine CcOs shows that a fairly large space between Glu^{242} and the O_2-reduction site. The space has no fixed water in the X-ray structures and is wide enough for allowing the structural changes in the side chain of Glu^{242} as shown in the inset. The D-pathway mechanism proposes that Glu^{242} functions as a branching point for pump-proton transfer and chemical-proton transfer and that the pump-protons are transferred to a proton-loading site (PLS) near the O_2-binding site much faster than the chemical-proton transfer to the O_2-reduction site. When the chemical proton arrives at the O_2-reduction site, the net positive charge increase at the O_2-reduction site drives the pump-proton release from the proton-loading site to the P-side phase electrostatically (1–3). This pump proton transfer to the PLS faster than that of the chemical-proton transfer to the O_2-reduction site avoids the direct transfer of the pump protons to the O_2-reduction site, which would dissipate the proton pump energy. This energy coupling mechanism is called “kinetic gating” (42).

One of the key structural elements of the D-pathway mechanism, the proton-loading site, has not been identified experimentally, though simulation analyses suggests the following three

candidates, the two propionate groups of heme a_3 and an imidazole group of one of the three histidine groups coordinated to the Cu_B (43). The X-ray structure of CcO indicates that space connecting Glu^{242} and the putative PLS, as described above, includes the space connecting Glu^{242} with the O_2-reduction site. It has been proposed that transiently introduced water molecules mediate the proton transfer from Glu^{242} to the PLS and to the O_2-reduction site (42). For the proton ejection to the P-side from the PLS (the proton pump) by the chemical proton which arrives after transfer of the pumping proton to the PLS, the pumping proton has to be kept on the PLS until the chemical proton arrives at the O_2-reduction site. For this pump-proton storage at the PLS, strong barrier for preventing the direct proton transfer from the PLS to the O_2-reduction site is necessary, since the proton transfer is highly exergonic (42). However, the X-ray structures of the space determined at various oxidation and ligand binding state reported thus far are essentially identical with that given in Fig. S1A. That is, no structural evidence suggesting the existence of the barrier in the space has been obtained (42).

Another unknown structure is the exit site for pumping protons from the PLS. Effective blockage of the protons in the P-side upon proton release to the P-side through the exit site is a prerequisite for effective energy transduction of CcO. Some candidate structures on the hydrophilic surface of CcO facing the P-side have been proposed based on simulation analyses (44, 45). However, these candidate structures in the X-ray structures do not show any redox-coupled structural change. Some hydration state changes have been proposed for preventing the proton back leak (45). Identification of the pump proton exit is critical for proving that the putative PLS keeps protons for pumping. In the X-ray structure of bovine heart CcO, the product water exit channel has been identified connecting the O_2-reduction site with the transmembrane surface of the CcO molecule (17). The location of the exit for the product water on the transmembrane surface strongly suggests that proton back leak from the P-side to the hydrophilic water exit channel is effectively blocked. The pumping protons on the putative PLS, which is close to the O_2-reduction site, is highly likely to be transferred through the product water channel to the exit on the transmembrane surface. The protons transferred to the transmembrane surface would be readily returned to the N-side by the membrane potential, and then to the N-side phase. The overall process of the proton transfer from the PLS to the N-side phase is highly exergonic. Thus, the location of the product water exit channel does not support the pumping proton transfer through Clu^{242} and the putative PLS.

A possible mechanism for the decoupling mutation, $Asn^{98}Asp$, has been proposed by an extensive simulation analysis for one of the proton pumping steps in the catalytic cycle, Pr→F (46). $Asn^{98}Thr$ and $Asn^{98}Ser$ also show an essentially identical phenotype, i.e., abolishment of the proton pump function without influencing the O_2-reduction activity. The simulation analyses for these mutants and the wild type CcO showed that the Asn gate including Asn^{98} and Asn^{80} sets a

large barrier for preventing reverse proton release from Glu^{242} to Asp^{91} at the entrance of the D-pathway (46) and that significant decreases in the barrier at the Asn gate by these mutations were observed giving enhanced reverse proton transfer rate from Glu^{242} to Asp^{91} (47). The calculated reverse proton transfer rate is significantly faster than the proton ejection (pump) rate from the PLS to the P-side, determined experimentally for the wild-type enzyme. Assuming that the decoupling mutation does not affect the proton ejection rate, since the mutation site is located sufficiently far from the PLS for affecting its function, it was concluded that the decoupling was induced by lowering the energy barrier at the Asn gate (47). However, the X-ray structure of the Asn^{98}Asp mutant enzyme showed a clear structural changes at Glu^{242}, suggesting that the function of the PLS, located near the Glu^{242}, is likely to be influenced by the mutation (48). Thus the above assumption must be evaluated experimentally. Furthermore, lowering the barrier for the reverse proton transfer is highly likely to enhance not only the reverse rate but also the forward rate. If the forward rate is sufficiently fast, the decrease in the barrier would not affect the proton pump function. However, the reference (47) does not provide the calculation results for the forward proton transfer which are necessary for evaluation of the simulation results reported.

The following mutational results provide perhaps the strongest experimental support for the D-pathway to transfer both pump and chemical protons: Asp^{91}Asn mutation abolishes the turnover CcO activity completely by blocking the F→O transition, while the Pr→F transition is not impaired. Asp^{91} is located at the entrance of the D-pathway as described in Fig. S1A. This result strongly suggests that the mutant CcO has two protons necessary for driving the Pr→F transition, one for protonation of the Pr form to provide the F-form and the other for protonation of the PLS which drives the A→Pr transition. (The A→Pr transition is coupled with a vectorial proton translocation assignable to that from Glu^{242} to the PLS and is blocked by the Glu^{242}Gln mutation (49).) Since Asn91 does not have proton, the third proton for the F→O transition is not available in this mutant enzyme. A double mutant Asp^{91}Asn/Tyr^{19}Phe impaired the Pr→F transition, without blocking the A→Pr transition, If the highly conserved Tyr^{19} reversibly accept one proton, as has been suggested (49), this double mutation result strongly support the proposal that the D-pathway keep two protons above Asp^{91}. However, it has been reported that the A→Pr transition is also blocked by a K-pathway mutation, Lys^{319}Met (50). This result strongly suggests an alternative interpretation as follows: Neither Glu^{242} nor Lys^{319} transfer pump-proton from Glu^{242} to the PLS while pump protons are transferred through a proton conducting pathway other than the D-pathway and both Gln^{242} and Met^{319} at D- and K-pathways, respectively, induce structural changes in the proton conducting pathway for pump-protons to block the proton transfer function of the pathway. Tyr^{19} does not keep chemical protons, but Phe^{19} abolishes the chemical proton transfer from Glu^{242} to the O_2-reductio site which drives the Pr→F transition (1, 3). The alternative interpretation has not been disproven yet. Although the D-pathway mechanism is

widely accepted in this field, careful examinations as described above indicate that various experimental works are still necessary for proving this mechanism.

2. H-pathway mechanism.

In contrast to the case of the D-pathway mechanism which has been constructed mainly by the mutational analyses for bacterial CcOs, an alternative proton-pump mechanism, H-pathway mechanism, has been proposed, based on various X-ray structural analyses for bovine heart CcO (1, 2). The H-pathway is composed of a hydrogen-bond network and a water channel as describe in Figs. 1 and 2D. The hydrogen-bond network is attached to heme *a* with two hydrogen bonds between its formyl and D-ring propionate groups (Fig. 1). This structure strongly suggests that pump-protons on the hydrogen-bond network of the H-pathway are expelled to the P-side through the hydrogen bond network electrostatically by the net-positive charges created on heme *a* upon the electron transfer to the O_2-reduction site. Back leakage of the pump-protons on the hydrogen-bond network is blocked by closing the water channel by eliminating one of the water cavities which enhance the mobility of the water (or hydronium ion) molecules inside the cavity as schematically described in Fig. 2D. The redox-coupled structural changes as described in Fig. 2A indicates an essentially irreversible redox-coupled proton release to the P-side, i.e., proton release to the P-side upon reduction of CcO. This structural change suggests effective avoiding of proton back leak from the P-side to the hydrogen-bond network of the H-pathway upon the proton ejection to the P-side. On the other hand, it has been established that the pump-protons are released upon oxidation of heme *a* (51-53). Thus, it has been proposed that some proton accepting groups keep the protons released from Asp^{51} until oxidation of heme *a* (14). However, this proposal has not been examined extensively.

The X-ray structures of all the intermediate forms of CcO involved in proton-pump indicate that the water channel of the H-pathway is able to collect protons from the N-side by the mobile waters only in the R-form, while in the other intermediates the water channel blocks proton-back leak form the hydrogen bond network of the H-pathway to the N-side by eliminating a water cavity of the water channel, as illustrated in Figs. 6 A and B (4, 8). The structural finding indicates that the four pump-protons are transferred in the R-form through the water channel up to above the water channel and kept for sequential donation to the hydrogen-bond network of the H-pathway. The structure of a large water cluster including a Mg^{2+} ion observed in high resolution X-ray structures of bovine heart CcO strongly suggests this pump-proton storage function as described in Fig. 1 (10). Namely, in each of the four proton-pump transitions (Pr→F, F→O, O→E, and E→R), one proton is released (from the water cluster) to the P-side coupled with uptake of one-water forming proton from the N-side. However, it has been shown that in each of the transitions, Pr→F and F→O, one proton release to the P-side is coupled with two proton release

from the N-side (54). This is most likely to be the case of the other two transitions, O→E, and E→R. This result strongly suggests that in each transition, in addition to one chemical proton, one pump proton is collected from the N-side. These pump protons are least likely to be stored in the Mg^{2+}-containing water cluster, as illustrated in Fig. 1, in each transition, since the water channel of the H-pathway blocks proton transfer through the channel as illustrated in Fig. 6A. Thus, this result suggests that CcO has a pool (or pools) for storage of these pump protons below the proton-blockage point of the water channel. These four pump protons collected in the pool are transferred to the Mg^{2+}-containing water cluster in the R-sate. Although an experimental result supports the existence of such a proton storage (55), any structural search for the proton pool has not been reported.

The necessity of protonation of His^{413} (Figs. 1 and 2D) in the water channel of the H-pathway for incorporation of water molecules into the water channel has been proposed by an atomic molecular dynamic simulation (56). The hydration is a prerequisite for formation of water-wire in the water channel for proton transfer. The X-ray structure of the fully-oxidized form at 1.8 Å resolution (PDB ID:1V54) shows that the main part of the water channel is not sufficiently hydrated for facile proton transfer. The microenvironment of His^{413} near the entrance of the water channel facing the N-side is unlikely to provide sufficiently low pH for protonation of the His^{413}. Thus, the simulation result and the X-ray structure suggest that the water channel is not able to transfer protons. However, the improved X-ray structures of CcO at 1.55 Å resolution or higher (PDB ID:5B1A, 5B1B, 5ZCQ, 5ZCP, 7COH, 7VUW, 7VVR, and 7YPY) show that the water channel is fully hydrated to provide the structure identical to that obtained by the above simulation, assuming protonation of His^{413}. Thus, these improved X-ray structures demonstrate that the water channel is active. The resolution, 1.8 Å, (PDB ID:1V54) is not sufficiently high for resolving the water molecules inside the water channel. This point has been mentioned previously (3, 23).

3. Mutational analyses of the H-pathway

Mutation analyses of the H-pathway of bovine heart CcO were conducted by using an expression system for bovine heart subunit I in HeLa cells (13, 14). As described in Fig, 2A, Asp^{51} is located at the exit of the hydrogen-bond network of the H-pathway and a peptide bond between Tyr^{440} and Ser^{441} is included in the network (Fig. 2A). The proton transfer through a peptide bound by forming an imidic acid intermediate (-C(OH)=N^{+}H-) has been well established (57). Both Asp^{51}Asn mutation and Ser^{441}Pro mutation provided an identical phenotype, abolishment of proton-pump function without influencing the electron transfer activity (13, 14). (The latter mutation, Ser^{441}Pro, blocks the formation of the imidic acid intermediate.) A closely similar phenotype was observed for a double mutation of the residues in the water channel, Val^{386}Leu/Met^{390}Trp, for elimination of the water cavity in the water channel (14). These mutation analyses strongly confirm the function of the H-pathway proposed based on the X-ray structural

analyses as described above.

The A-type bacterial CcOs have a proton conducting pathway analogous to the H-pathway, although some critical residues, such as Asp^{51}, is not conserved in the bacterial CcOs. Arg^{38} located at the N-side end of the H-pathway of the bacterial CcO (see Fig. 1) was replaced with methionine (58). This mutation significantly decreased the turnover rate without influencing the proton pumping efficiency. It was claimed that this mutation disproved the proton pumping function of the H-pathway, since this mutation broke the hydrogen bond network of the H-pathway. However, the methionine side chain replaced with that of arginine would not break the hydrogen bond network of the H-pathway and the formyl group of heme *a* would interact with the hydrogen bond network of the H-pathway located nearby to drive the proton pump. Thus, the decrease in the overall activity by this mutation suggests the involvement of the hydrogen-bond network of the H-pathway in the proton pump function. Tyr^{371}Phe mutant included in the hydrogen bond network of the H-pathway as described in Fig. 2A was as active as the wild type (59). Thus, it was proposed that this result proved that the hydrogen-bond network was not critically involved in the catalytic turnover. However, Phe^{371} would introduce a fixed water at the position of the Tyr^{371}OH, retaining the hydrogen-bond network. Point mutations of several residues in the water channel of the bacterial H-pathway with less bulkier residues did not show any significant influence on the phenotype function (59). These mutations would not influence the movements of the water molecules in the water channel. Thus, mutation results for the bacterial H-pathway reported thus far provide any conclusive evidence disproving the involvement of the H-pathway in the proton pumping function of the bacterial CcOs. It is noteworthy that, in general, only if a mutation of a residue has a clear effect on the function of a functional protein, the mutational analysis can prove critical involvement of the residue in the function of the protein.

The function of the H-pathway of mitochondrial CcO of a yeast, *Saccharomyces cerevisiae*, were examined by a newly developed method for mitochondrial DNA mutagenesis (60). The H-pathway mutations, Gln^{411}Leu, Gln^{413}Leu, Ser^{382}Ala, Ser^{458}Ala, Ser^{455}Ala, and Ser^{52}Asp, provided no significant influence on the enzyme function of the yeast CcO. From this mutational results, it was concluded that the H-pathway of the yeast CcO was not involved in the proton pumping function (60). Although Gln^{411}Leu, Gln^{413}Leu, Ser^{458}Ala, and Ser^{455}Ala are located in or near the water channel of the H-pathway, the structural changes in these mutations are too small to block the water channel, in contrast to the case of the double mutation of bovine CcO, Val^{386}leu/Met^{390}Trp as described above (14). Ser^{382} is hydrogen-bonded to the hydoxyfarnesyl ethyl group of heme *a* to eliminate a water cavity of the H-pathway to block the water access to the hydrogen-bond network of the H-pathway as described above (Fig. 6A). However, an energy minimization analysis indicates that the structural change induced by

$Ser^{382}Ala$ mutation is too small to influence the structural changes in the Ser^{382}-including region detectable in the wild type CcO (3). Ser^{52} is located in a fairly hydrophilic environment facing the P-side phase. The $Ser^{52}Asp$ mutation is unlikely to influence the function of the H-pathway. However, from these results, it was concluded that the H-pathway of the yeast CcO was not involved in the proton pump function (60), as in the case of the bacterial H-pathway mutants as described above.

Compared with structures of the K- and D-pathways of the A-type CcO, the diversity in the structure of the H-pathway is significantly higher. A typical example would be that Asp^{51} is conserved only in animal kingdom. The A-type CcOs of plants (including yeast) and bacteria do not have Asp^{51}. The simplest interpretation for the diversity is that the H-pathway does not have the proton pump function, since the structure facilitating such a physiologically important function as the proton pump must be completely conserved. However, it is also possible that the diversity is induced by evolutional adaptation of the energy requirement of each species. Essentially complete conservation of Asp^{51} in animal kingdom strongly suggests this possibility (3).

4. Conclusion

As described above, at present, both the D- and H-pathway mechanisms coexist in this research field (1–3). Perhaps, one of the most critical experimental works for proving the D-pathway mechanism would be identification of the structure giving a barrier for complete prevention of spontaneous pump-proton leak from the PLS to the O_2-reduction site. For proving the H-pathway mechanism, it is absolutely necessary for finding the pool for keeping the pump-protons below the proton-blockage point of the water channel.

Supporting Text 2.

Preparation of the cyanide-bound CcO crystals.

1. CNox crystals. Isomorphous crystals of the fully-oxidized and fully reduced forms were prepared as described in a previous paper (8). The CNox crystals were prepared by treating the fully-oxidized CcO crystals with cyanide for 3 days with frequent exchanges of the freshly prepared cyanide solution, since the fully-oxidized form of CcO receives cyanide fairly slowly and cyanide in aqueous solution is not very stable. The complete saturation of the cyanide-binding site was monitored by the absorption spectrum of the Soret band at 428 nm.

2. CNmv crystals. For preparation of the CNmv crystals, the CNox crystals were treated at 4˚C with 2.5 mM dithionite anaerobically in the presence of a glucose-glucose oxidase-catalase system for scavenging trace amount of O_2 leaked to a treatment chamber (a hollow slide covered with a cover slide without gas space). In the crystals, significant reduction of Fe_{a3}^{3+}-CN^- to Fe_{a3}^{2+}-CN^- was observed with its characteristic absorption band at 590 nm (61) though the reduction is

much slower than reduction of the other metal sites. Absorption spectral changes in the α-band region were monitored during the dithionite reduction for stopping the reduction just before an absorbance increase at 590 nm, using a nitrogen stream at 100K, immediately after attainment of the maximum stable band at 604 nm, indicating complete heme *a* reduction, before the absorption increment at 590 nm due to formation of the Fe_{a3}^{2+}-CN^- started (61). The effective flash cooling with a nitrogen stream at 100 K for stopping the absorbance increase was confirmed by the absorption spectrum of the frozen crystals before X-ray diffraction experiments.

3. CNmv/CNred crystals. The CNred crystals were prepared by treating the fully-oxidized form crystals with 2.5 mM dithionite and 5 mM cyanide under anaerobic conditions applied for preparation of the CNmv crystals as described above. For comparison, the fully reduced form crystals were prepared under the identical conditions without adding cyanide. Crystals with a closely similar size were used for minimizing crystal size influence on the absorption spectral characteristics of CcO in the crystal. The α-band absorption spectra of the CNred crystals obtained under the present conditions are given in Fig. S2. It took about 5 min to attain these spectra and no further spectral change was detectable. The clear 590 nm band, as intense as the 604 nm band in the CNred crystal, indicates the complete formation of the CNred in the crystals under the present conditions (61). After the stable spectrum was attained, each crystal was flash-cooled on a small loop for X-ray diffraction experiments by a nitrogen stream at 100K. This flash-cooling procedure induced a significant decrease in the 590 nm band as shown in Fig. S2, consistent to the previous report (18). This spectral change suggests that some part of the CNred in the crystals is oxidized to the CNmv by a limited amount of O_2 leaked during the flash-cooling procedure as follows; because of the much weaker affinity of cyanide to Fe_{a3}^{2+} than that of O_2, a significant amount of the cyanide bound at Fe_{a3}^{2+} is replaced with O_2 to form the O_2-bound fully reduced form which would be readily oxidized to the O-form. Then, the O-form receives cyanide and the residual dithionite in the mother liquor provides the CNmv which does not have the 590 nm peak. By using crystals with a similar size, fairly consistent absorption spectra were obtained for the flash-cooled crystals. However, the conditions for complete avoidance of the O_2 leak during the flash-cooling were not established. Reliable quantitative estimation of the ratio of the CNred to the CNmv by the absorption spectral results is impossible since the extinction coefficients of these CcO species in the crystals are yet to be determined. In this paper, the frozen crystal prepared as described above is designated as the CNmv/CNred crystal. No significant X-ray irradiation effect is detectable on the absorption spectra of crystals taken after the X-ray diffraction experiments.

Supporting Text 3.

Structural determination of a model with a singular structure

The structural model with the fully-oxidized form structure for any part of the

molecule was superimposed well on the MR/DM maps of the CNox data set. The model with structure of the fully reduced form is superimposed well on the MR/DM map of the CNmv data set, except for residues 380–385 of helix X of subunit I which provided the fully-oxidized form structure. The fully reduced form structure except for heme a_3 was superimposed well on the MR/DM map of the CNmv/CNred data set. A model structure of heme a_3 of the CNmv/CNred data set was built on the map by translating the fully reduced form structure. Structure refinements were performed by alternating round of model building with program COOT (62) and restraint refinement with program Phenix.refine (63). In the initial stage of the refinements of these data sets, multiple structures were not included in the regions around Asp^{51} and Ser^{382} of subunit I and the hydroxyfarnesyl ethyl groups of heme *a* and heme a_3. The refinement of the CNox data set converged to the fully-oxidized form structure in any part of the molecule. The CNmv data set converged to the fully reduced form structures in the residue 48–55 region and the hydroxylfarnesyl ethyl group of heme *a*, and to the fully-oxidized form structure in residues 380–385 of helix X. The converged structure of heme a_3 designated as the CNmv structure is an intermediate structure between the fully-oxidized form and fully reduced form structures. The CNmv/CNred data set converged to the fully reduced form structure in residues 48–55 and hydroxylfarnesyl ethyl group of heme *a* as in the case of the CNmv data set. During the process for the refinement of the CNmv/CNred data set starting from the fully reduced form structure of helix X, the helix X structure converged to a new structure designated as the CNred structure. Residues 27–47 of subunit II of the CNmv/CNred data set converged to a new structure designated as the CNred structure.

Supporting Text 4.

Multiple structure identification

The same procedures as the previous paper (8) were applied for detecting the coexistence of multiple structures. The *B*-factors of the main chain portions of subunit I were plotted and the *Fo-Fc* maps of regions, where unusually high *B*-factor values were distributed, were calculated for detection of multiple structures. Distributions of *B*-factors of residues 40–70 and 370–395 of subunit I, where clear redox coupled conformational changes are observed between the fully-oxidized form and the fully reduced form, are shown for the CNox, CNmv, and CNmv/CNred data sets, together with the fully-oxidized form data (PDB 5B1A) in Fig. S3. These high *B*-factor values could be induced either by the existence of an additional structure as a minor component or by high thermal motions. If a minor component exists in an electron density map calculated as a singular structure, the *Fo-Fc* map against the major component structure would provide positive and negative electron densities corresponding to the minor and major component structures, respectively. Figure S4 shows the *Fo-Fc* maps of the CNox data set

around Asp^{51} of subunit I with the fully-oxidized form structure (in A) and the fully reduced form structure (in B). A negative peak in the map with the fully-oxidized form structure as marked by an arrow is located at the position of a carbon atom of Asp^{51} of the fully-oxidized form (Fig. S4A), while two positive peaks are detectable at the positions of the carbon atoms of Asp^{51} and Gln^{52} of the fully reduced form structure as marked by the arrows (Fig. S4B). This *Fo-Fc* map strongly suggests that coexistence of the fully reduced form structure as a minor component induces the unusually high *B*-factor values in the region of residues 40–70 of the CNox data set. Similar *Fo-Fc* map analyses for the CNmv data set revealed that in residues 380–385 of helix X (Figs。 S5A and B) and the hydroxyfarnesyl ethyl group (Figs. S5C), the fully reduced form structure and the fully-oxidized form structures coexist as the major and minor components, respectively. For the CNmv/CNred data set, coexistence of minor fully-oxidized form structure and major fully reduced form structure was detectable in the hydroxyfarnesyl ethyl group of heme *a* as in the case of the CNmv data set (Figs. S6A). Coexistence of the CNmv structure in the CNred structure were detectable in heme a_3 (Fig. S6B). In the region of the residue 27–47 of subunit II, the CNmv/CNred data set showed coexistence of the structure identical to that of the CNmv form (Fig. S6C). The CNmv form has the region of the residue 27–47 identical to the fully-oxidized form. The CNred structure and the fully-oxidized form structure coexist in this region of the CNmv/CNred data set (Fig. S6C). In this figure, the area where positive peaks corresponding to the fully-oxidized form structure are detectable is marked by a dotted loop.

By inspecting *Fo-Fc* maps, we assigned coexisting structures qualitatively, as described above. For quantitative evaluation of the content of the coexisting structure, the refinements were performed for different contents of two components searching the content ratio of the two components that yield an identical average *B*-factor for both component structures. By determination of the ratio, the content of each component in a multiple structure can be estimated, since all of the component structures are highly likely to have an identical average *B*-factor value. Figure S7A shows the effect of content of the fully-oxidized form structure on ΔB, the difference between the average *B*-factors of the two components, in the structure of the region of residues 380–385 of the CNmv data set, determined for monomers A and B, illustrated with the blue and red plots, respectively. The two plots which close the horizontal axis at 0.55 indicate that the content ratio of the fully-oxidized form structure to that of the fully reduced form structure is 0.55/0.45 for both monomers. The ΔB plots for the hydroxyfarnesyl ethyl group of heme *a* given in Fig. S7B show that the content ratio of the fully-oxidized form structure to the fully reduced from structure is 0.25/0.75 and 0.23/0.77 for monomers A and B, respectively. The ΔB plots for residues 48–55 of subunit I of CNox data set,

given in Fig. S8 suggest that the ratio for the fully-oxidized form structure/the fully reduced form structure is 0.90/0.10 for both monomers. For the CNmv/CNred data set, the fully reduced form structure/the CNred structure for the residues 380–385 of helix X is 0.30/0.70 and 0.40/0.60 for monomers A and B, respectively (Fig. S9). An *Fo-Fc* map was calculated with the structure in which the region with residues 380-385 of the helix X consisted of the fully reduced form structure and the CNred structures, each with the estimated occupancies (Figure S10). Fig. S10A shows the negative peaks with atomic models of the fully reduced form and CNred structures, while positive peaks with the fully-oxidized form structure are given in Fig. S10B. The arrows in both panels indicate the coexistence of the fully-oxidized form as another minor component in the region with residues 380–385 of the helix X. Ratios of three components determined by the final refinement are given below (Supporting text 6. 3.). The fully-oxidized form structure/the fully reduced form structure ratios for the hydroxyfarnesyl ethyl group of heme *a* in the CNmv/CNred data set were 0.25/0.75 and 0.30/0.70 for monomers A and B, respectively (Fig. S11A). The structure of residues 27–47 region of subunit II showed the fully-oxidized form structure/the CNred structure ratios of 0.42/0.58 and 0.65/0.35 for monomers A and B, respectively (Fig. S11B). The CNmv structure /the CNred structure ratios in heme a_3 structure of the CNmv/CNred data set were 0.44/0.56 and 0.66/0.34 for monomers A and B, respectively (Fig. S11c).

Supporting Text 5

Determination of the structure of O_2-reduction site and final structural refinements

For the third step, at first, providing multiple structures determined by the second step, structure refinements were carried out over several rounds without any ligand in the O_2 reduction site. CN ligands were located in the *F*o-*F*c maps of the CNox, CNmv, and CNmv/CNred data sets (Figs. S12A, B, and C). The structures of the CNox and CNmv data sets were refined without any structural restraints for CN^- ligands at first, and the resultant structures of CN^- ligands were applied to restraint conditions for the CN^- ligands in the further refinements. The structure of the CNmv/CNred data set with the CN^- ligand of heme a_3 of the CNred structure with 50% occupancy was refined and another CN^- ligand of the CNmv heme a_3 was located in the *F*o-*F*c map (Fig. S12D). Both the CN^- ligands were included and restrained as in the cases of the CNox and CNmv data **set**s in the further refinement.

Ratios of multiple components were readjusted in the further refinements. Arg^{438} and Glu^{198} of the CNox **data set** represented the fully-oxidized form structure, and those of the CNmv and CNmv/CNred crystals showed the fully reduced form structure. Both the CNox and CNmv data **set**s showed an essentially identical structure for His^{290}, which is different from that of the CNmv/CNred data set. Assigning a multiple structure including that of the CNmv data set and a

new structure to His^{290}, the structure of CNmv/CNred data **set** was refined by several rounds. Structures of His^{290} were converged to that detectable in the CNmv data set and the new structure, designated as the CNred structure of His^{290}. Statistics of the final stage of the refinements are given in Table S2. A summary of the present X-ray structural determination is given in Table S3.

Supporting Text 6.

The final X-ray Structures determined by the present analyses.

1. CNox.

Most parts of the structure of the CNox, determined as described above, consisted of the same structures as the fully-oxidized form. However, residues 48–55 of subunit I contained 10% of the structure closely similar to the fully reduced form. An infrared analysis of the reaction between the fully-oxidized form and cyanide under essentially identical conditions strongly suggests that the 10% reduction of CcO is induced by a spontaneous reduction of CcO by cyanide (19). Thus, the CNox is unlikely to have the fully reduced form structure in this region. The atomic distances in the final ligation structures (Fig. 3), averaged for the monomers A and B are as follows, 4.87 Å and 4.85 Å for Fe_{a3}-Cu_B of the CNox and the fully-oxidized form, respectively, 2.14 Å for Fe_{a3}-C and 2.21 Å for Cu_B-N for the CNox, and 2.26 Å for Fe_{a3}-O1 and 2.16 Å for Cu_B-O2 for the fully-oxidized form, where O1 and O2 denote the oxygen atoms of a peroxide anion ligated to Fe_{a3} and Cu_B, respectively (Fig. 3 inset). These structures confirm the previous conclusion from the X-ray structure determined at 2.0 Å resolution (17).

2. CNmv.

The heme a_3 of the X-ray structure of the CNmv was located at a position halfway between those of the fully-oxidized (green) and the fully reduced forms (red) (Fig. 4A). The *B*-factors of the heme a_3 in the final X-ray structure of the CNmv were not significantly higher than those of the heme *a*. The averaged *B*-factors for hemes *a* and a_3 were 20.9 ± 1.3 $Å^2$ and 22.8 ± 1.7 $Å^2$, respectively, for monomer A and 24.7 ± 1.6 $Å^2$ and 24.2 ± 1.2 $Å^2$, respectively, for monomer B. Thus, heme a_3 of the X-ray structure of the CNmv is assignable as a singular structure. As given in Fig. S13, the *Fo-Fc* map of the X-ray structure of the CNmv (left panel) provided smooth residual density in the heme a_3 significantly lower than those of two hypothetical models, in each of which the heme a_3 structure was replaced by heme a_3 of the fully reduced form (center panel) or by that of the fully-oxidized form (right panel). Thus, significant structural differences between them are obvious. The averaged atomic displacements of porphyrin rings were 0.20 Å with r.m.s.d. of 0.06 Å between the CNmv and the fully-oxidized form, 0.27 Å with r.m.s.d. of 0.04 Å between the CNmv and the fully reduced-form, and 0.32 Å with r.m.s.d. of 0.09 Å between the fully-oxidized and fully reduced forms.

The coordination structure of the CNmv in the O_2 reduction site is illustrated in yellow and superimposed with the coordination structure of the fully-oxidized CcO in green in Fig. 4B.

The cyanide ligand of the CNmv crystal coordinated to Fe_{a3} in an essentially straight fashion (Fig. 4B). The angles averaged for the two monomers for the three atoms, Fe_{a3}-C-N and C-N-Cu_B for the CN^- ligand of CNmv crystal were 163.4° and 168.7°, respectively. The atomic distances in the O_2 reduction site, averaged for the results of the two monomers were 4.96 Å for Fe_{a3}-Cu_B, 1.90 Å for Fe_{a3}-C, and 2.02 Å for Cu_B-N. The Fe_{a3}-Cu_B distance is longer than that of the CNox (4.85 Å) and shorter than that of the fully reduced form (5.18 Å). These location and ligation structure of the heme a_3 are detectable only in the CNmv and undetectable in both the fully-oxidized and fully reduced forms.

3. CNred.

Figures S14A and B illustrate the final structures of the heme a_3 and the cyanide-liganded structure of the CNmv/CNred data set, respectively. Obviously, each structure is composed of two components. In Figs. S14C and D, the heme a_3 and cyanide ligand structures of the CNmv crystal (green-colored) are superimposed to those of the CNmv/CNred data set (yellow-colored), shown in Figs. S14A and B. The green-colored structures coincide very well with one of the component structures of the CNmv/CNred data set. Thus, the other component structure of heme a_3 is assignable to that of the CNred (*CNred* in Table 1). This structure is different from both those of the fully-oxidized form and fully reduced form. The content ratio of the CNred to the CNmv, averaged for the two monomers was 57/43 (Table 1). The existence of the CNmv in the O_2 reduction site of the CNmv/CNred crystals, shown qualitatively by the absorption spectroscopic observation as described above, was confirmed by this X-ray structural analysis quantitatively.

The previous analysis for the CNmv/CNred data set was performed without taking the coexistence of the CNmv in the CNred crystal into account, although the absorption spectrum of the frozen crystals suggested incomplete formation of the cyanide-bound fully reduced form (i.e., lower intensity of the 590 nm peak of the frozen crystals compared with that at ambient temperature) (18). Analysis of the present X-ray diffraction data sets, ignoring the coexistence of the CNmv, provides essentially identical structures reported in the previous paper (18).

Figure S15 shows the final structure of the peptide back bone of residues 377–383 of subunit I of the CNmv/CNred data set, giving three component structures. The occupancy ratio of the three components, designated as A, B, and D in the figure, averaged for the two monomers, was 25/26/49 (Table S3). The 25/26 % portion is assignable to the CNmv, which contains essentially equal amount of the fully-oxidized form structure and the fully reduced form structure in this region, giving the ratio of 58/42 for both monomers (Tables 1 and S3). The content ratio, 25/26/49, averaged for the two monomers is consistent to the CNmv/CNred structure ratio, 57/43, averaged for the two monomers, determined from the cyanide-ligation structure (Table S3).

Structures of helices VIII and IX of the CNmv/CNred data set, suggesting small structural differences from the fully reduced form in the previous paper (18), had higher *B*-factor

values than those of surrounding regions in the present structure. Residues 27–47 of subunit II of the CNmv/CNred data set shown in Fig. S16A shows existence of two structures, one of which is superimposed well on that of the CNmv (green-colored) (Fig. S16B). The content ratio of the two structures in the CNmv/CNred data set, averaged for the two monomers in the region of the residues 24–47 of subunit II, 55/45 (= (45/55 + 65/35)/2), is consistent to those values, 57/43 (= (52/48 + 62/38)/2) and 51/49 (= (20/24/56 + 30/28/42)/2), determined from the structures of the heme a_3 and the residues 380-385, respectively (For digits in the parentheses, see the CNmv/CNred line in Table S3.). Thus, the structure different from that of the CNmv is assignable to the structure of the CNred. As shown in Fig. S16, including the heme a_3 structures in the final structure of the CNmv/CNred data set, the translational movement of the heme a_3 upon reduction of Fe_{a3}^{3+}-CN^- (i.e., transition from the CNmv to the CNred) triggers the structural transition in residues 27–47 of subunit II.

4. The residues 27-45 of the two azide-bound fully-oxidized form.

The present recalculation for the data set for the azide-bound fully-oxidized CcO crystals prepared at 20 mM azide (PDB 5Z84), revealed that 10% and 90% of the one and two azide-bound fully-oxidized forms, respectively, coexisted, giving the content ratios of the fully-oxidized form structure/the CNred structure of 0.55/0.45 for both heme a_3 and the residues 377–385 of helix X of subunit I and of 0.61/0.39 for residues 27–47 of subunit II. Because the one azide-bound form provides the fully-oxidized form structure in all these structural regions (18), the two azide-bound form provides the fully-oxidized form structure/the CNred structure ratio of 0.45/0.45 for both heme a_3 and residues 377–385 of helix X of subunit I and of 0.51/0.39 for residues 27–47 of subunit II. The recalculated structure given in Fig. S18 superposes well with the structure of the CNmv/CNred data set, which is composed of the structures of the CNred and the CNmv. However, the structure of the CNmv in the region given in Fig. S18 is closely similar to that of the fully-oxidized form. Thus, within the experimental accuracy, the recalculation result indicates that one of the hemes a_3 of the two azide-bound form, located at the position identical to that of the CNred, induces the CNred structure in residues 27–47 of subunit II. That is the case for the residues 380–385 of helix X of subunit I. These results suggest that these two structures are induced by the location of the heme a_3.

Table S1. Statistics of intensity data collection of three types of the cyanide-bound CcO crystals

Crystal	CNox	CNmv	CNmv/CNred
Beam line		SPring-8 BL44XU	
Beam sizes (μm)		50 (v) × 30 (h)	
Space group	$P2_12_12_1$	$P2_12_12_1$	$P2_12_12_1$
Number of crystals	12	8	10
Number of images	935	1,020	2,360
Cell constants (Å)			
a	182.08	182.31	181.67
b	204.48	204.71	203.75
c	177.68	177.38	177.68
Resolution (Å)	135.98-1.60 (1.61-1.60)	136.18-1.60 (1.61-1.60)	135.60-1.45 (1.46-1.45)
Observed reflections	14,551,688	13,176,314	25,565,863
Independent reflections	859,689 (21,340)	862,696 (21,415)	1,155,078 (28,677)
Completeness (%)	100.0 (100.0)	100.0 (100.0)	100.0 (100.0)
Redundancy	16.9 (15.7)	15.3 (12.7)	22.1 (13.9)
R_{merge}	0.091	0.091	0.082
R_{pim}	0.023 (0.339)	0.023 (0.398)	0.016 (0.383)
$I/\sigma(I)$	43.68 (3.50)	45.11 (2.79)	58.5 (3.27)

Numbers in parentheses are given for the highest resolution shell.

Table S2. Structure refinement statistics of three types of the cyanide-bound CcO data sets

Crystals	CNox	CNmv	CNmv/CNred
Resolution (Å)	40.0~1.60	40.0~1.65	40.0~1.45
	(1.62~1.60)	(1.67~1.65)	(1.47~1.45)
Number of reflections	858,998	786,012	1,146,769
in work set	(25,182)	(24,646)	(35,917)
Number of reflections	43,190	39,530	57,645
in test set	(1,278)	(1,316)	(1,904)
R_{work}	0.1535	0.1523	0.1620
	(0.2434)	(0.2377)	(0.3027)
R_{free}	0.1754	0.1756	0.1817
	(0.2717)	(0.2595)	(0.3124)
Number of non-hydrogen atoms			
Total	34,951	34984	35,109
Proteins	29,064	29,461	29415
Lipids	1512	1485	1478
Detergents	561	664	684
Waters	2805	3000	3327
Ethylene glycol	995	360	200
Phosphate	10	10	5
Ligands	4	4	8
R.m.s. deviations			
Bonds (Å)	0.018	0.018	0.018
Angles (°)	1.898	1.884	1.965
DPI (Å)*	0.054	0.058	0.046
Ramachandran statistics (%)			
Favoured	96.92	97.22	97.21
Allowed	2.67	2.27	2.27
Outliers	0.40	0.52	0.52
Clashscore	8.34	6.98	7.90
Averaged B-factor ($Å^2$)			
Overall	38.19	39.39	40.75
Protein (A)	31.86	33.95	34.77
Protein (B)	37.30	38.69	40.09
Others	55.81	55.39	57.42

*DPI is a diffraction-component precision index, an approximate value of standard deviation of atomic position in protein crystal (63).

Table S3. Summary of X-ray structural analyses

Crystals (PDB_ID)		Asp[51†] *ox*/*red*	Arg[438] *ox* /*red*	Glu[198] *ox* /*red*	heme *a*[‡] *ox*/*red*	Ser[382§] *ox*/*red*/*CNred*	heme a_3[‖] *ox*/*red*/*CNmv*/ *CNred*	27-47(II)[¶] *ox* /*CNred*
fully-oxidized (5B1A)	A*	100/0	100/0	100/0	100/0	100/0/0	100/0/0/0	100/0
	B*	100/0	100/0	100/0	100/0	100/0/0	100/0/0/0	100/0
fully reduced (5B1B)	A*	0/100	0/100	0/100	26/74	34/66/0	0/100/0/0	100/0
	B*	0/100	0/100	0/100	23/77	35/65/0	0/100/0/0	100/0
CNox (7VUW)	A*	90/10	100/0	100/0	100/0	100/0/0	100/0/0/0	100/0
	B*	90/10	100/0	100/0	100/0	100/0/0	100/0/0/0	100/0
CNmv (7VVR)	A*	0/100	0/100	0/100	28/72	58/42/0	0/0/100/0	100/0
	B*	0/100	0/100	0/100	28/72	58/42/0	0/0/100/0	100/0
CNmv/ CNred (7W3E)	A*	0/100	0/100	0/100	25/75	20/24/56	0/0/52/48	45/55
	B*	0/100	0/100	0/100	30/70	30/28/42	0/0/62/38	65/35

A* and B* denote one of two monomers in the asymmetric unit of the crystal. Figures indicate percentage of each component. *ox* denotes the structure detectable in the fully-oxidized form. *red* denotes the structure detectable in the fully reduced form but not in the fully-oxidized form. *CNred* denotes the structure detectable in the CNred but undetectable in both the fully-oxidized and fully reduced forms. *CNmv* denotes the location and structure of heme a_3 detectable only in the CNmv. [†]The residues 45-55 of subunit I. [‡]The hydroxyfarnesyl ethyl group of heme *a*. [§]The residues 380-385 regions of helix X. [‖]Location of heme a_3. [¶]The residues 27-47 of subunit II.

Supporting References

41. Pawate, A. S., Morgan, J., Namslauer, A., Mills, D., Brzezinski, P., Ferguson-Miller, S., and Gennis, R. B. (2002) A mutation in subunit I of cytochrome oxidase from *Rhodobacter sphaeroides* results in an increase in steady-state activity but completely eliminates proton pumping. *Biochemistry*. **41**, 13417–13423

42. Popović, D. M., and Stuchebrukhov, A. A. (2012) Coupled electron and proton transfer reactions during the O→E transition in bovine cytochrome *c* oxidase. *Biochim. Biophys. Acta*. **1817**, 506–517

43. Popović, D. M. (2013) Current advances in research of cytochrome *c* oxidase. *Amino Acids*. **45**, 1073–1087

44. Popović, D. M., and Stuchebrukhov, A. A. (2005) Proton exit channels in bovine cytochrome *c* oxidase. *J. Phys. Chem. B*. **109**, 1999–2006

45. Cai, X., Haider, K., Lu, J., Radic, S., Son, C. Y., Cui, Q., and Gunner, M. R. (2018) Network analysis of a proposed exit pathway for protons to the P-side of cytochrome *c* oxidase. *Biochim. Biophys. acta. Bioenerg.* **1859**, 997–1005

46. Liang, R., Swanson, J. M. J., Peng, Y., Wikström, M., and Voth, G. A. (2016) Multiscale simulations reveal key features of the proton-pumping mechanism in cytochrome *c* oxidase. *Proc. Natl. Acad. Sci. U. S. A.* **113**, 7420–7425

47. Liang, R., Swanson, J. M. J., Wikström, M., and Voth, G. A. (2017) Understanding the essential proton-pumping kinetic gates and decoupling mutations in cytochrome *c* oxidase. *Proc. Natl. Acad. Sci. U. S. A.* **114**, 5924–5929

48. Dürr, K. L., Koepke, J., Hellwig, P., Müller, H., Angerer, H., Peng, G., Olkhova, E., Richter, O.-M. H., Ludwig, B., and Michel, H. (2008) A D-pathway mutation decouples the *Paracoccus denitrificans* cytochrome *c* oxidase by altering the side-chain orientation of a distant conserved glutamate. *J. Mol. Biol.* **384**, 865–877

49. Belevich, I., Gorbikova, E., Belevich, N. P., Rauhamäki, V., Wikström, M., and

Verkhovsky, M. I. (2010) Initiation of the proton pump of cytochrome *c* oxidase. *Proc. Natl. Acad. Sci. U. S. A.* **107**, 18469–18474

50. Lepp, H., Svahn, E., Faxén, K., and Brzezinski, P. (2008) Charge transfer in the K proton pathway linked to electron transfer to the catalytic site in cytochrome *c* oxidase. *Biochemistry*. **47**, 4929–4935

51. Capitanio, N., Capitanio, G., Boffoli, D., and Papa, S. (2000) The proton/electron coupling ratio at heme *a* and Cu_A in bovine heart cytochrome *c* oxidase. *Biochemistry*. **39**, 15454–15461

52. Capitanio, N., Capitanio, G., Minuto, M., De Nitto, E., Palese, L. L., Nicholls, P., and Papa, S. (2000) Coupling of electron transfer with proton transfer at heme *a* and Cu_A (redox Bohr effects) in cytochrome *c* oxidase. Studies with the carbon monoxide inhibited enzyme. *Biochemistry*. **39**, 6373–6379

53. Forte, E., Barone, M. C., Brunori, M., Sarti, P., and Giuffrè, A. (2002) Redox-linked protonation of cytochrome *c* oxidase: the effect of chloride bound to Cu_B. *Biochemistry*. **41**, 13046–13052

54. Faxén, K., Gilderson, G., Adelroth, P., and Brzezinski, P. (2005) A mechanistic principle for proton pumping by cytochrome *c* oxidase. *Nature*. **437**, 286–289

55. Zaslavsky, D., Sadoski, R. C., Rajagukguk, S., Geren, L., Millett, F., Durham, B., and Gennis, R. B. (2004) Direct measurement of proton release by cytochrome *c* oxidase in solution during the F-->O transition. *Proc. Natl. Acad. Sci. U. S. A.* **101**, 10544–10547

56. Sharma, V., Jambrina, P. G., Kaukonen, M., Rosta, E., and Rich, P. R. (2017) Insights into functions of the H channel of cytochrome *c* oxidase from atomistic molecular dynamics simulations. *Proc. Natl. Acad. Sci. U. S. A.* **114**, E10339–E10348

57. Perrin, C. L. (1989) Proton exchange in amides: Surprises from simple systems. *Acc. Chem. Res.* **22**, 268–275

58. Jasaitis, A., Backgren, C., Morgan, J. E., Puustinen, A., Verkhovsky, M. I., and Wikström, M. (2001) Electron and proton transfer in the arginine-54-methionine mutant of

cytochrome *c* oxidase from *Paracoccus denitrificans*. *Biochemistry*. **40**, 5269–5274

59. Lee, H. M., Das, T. K., Rousseau, D. L., Mills, D., Ferguson-Miller, S., and Gennis, R. B. (2000) Mutations in the putative H-channel in the cytochrome *c* oxidase from *Rhodobacter sphaeroides* show that this channel is not important for proton conduction but reveal modulation of the properties of heme *a*. *Biochemistry*. **39**, 2989–2996

60. Maréchal, A., Xu, J.-Y., Genko, N., Hartley, A. M., Haraux, F., Meunier, B., and Rich, P. R. (2020) A common coupling mechanism for A-type heme-copper oxidases from bacteria to mitochondria. *Proc. Natl. Acad. Sci. U. S. A.* **117**, 9349–9355

61. Hill, B. C., and Marmor, S. (1991) Photochemical and ligand-exchange properties of the cyanide complex of fully reduced cytochrome *c* oxidase. *Biochem. J.* 279 (Pt 2), 355–360

62. Emsley, P., Lohkamp, B., Scott, W. G., and Cowtan, K. (2010) Features and development of Coot. *Acta Crystallogr. Sect. D Biol. Crystallogr.* **66**, 486–501

63. Afonine, P. V., Grosse-Kunstleve, R. W., Echols, N., Headd, J. J., Moriarty, N. W., Mustyakimov, M., Terwilliger, T. C., Urzhumtsev, A., Zwart, P. H., and Adams, P. D. (2012) Towards automated crystallographic structure refinement with *phenix.refine*. *Acta Crystallogr. Sect. D Biol. Crystallogr.* **68**, 352–367
